# Ubiquitin modification in the regulation of tumor immunotherapy resistance mechanisms and potential therapeutic targets

**DOI:** 10.1186/s40164-024-00552-0

**Published:** 2024-08-30

**Authors:** Zihang Hong, Furong Liu, Zhanguo Zhang

**Affiliations:** 1 Hepatic Surgery Center, Tongji Hospital, Tongji Medical College, Hubei Province for the Clinical Medicine Research Center of Hepatic Surgery, Huazhong University of Science and Technology, 1095 Jiefang Avenue, Wuhan, 430030 Hubei China; 2 Hubei Key Laboratory of Hepato-Pancreato-Biliary Diseases, Wuhan, 430030 Hubei China; 3 Key Laboratory of Organ Transplantation, NHC Key Laboratory of Organ Transplantation, Key Laboratory of Organ Transplantation, Ministry of Education, Chinese Academy of Medical Sciences, Wuhan, China

**Keywords:** Cancer immunotherapy, Ubiquitin, Drug resistance

## Abstract

Although immune checkpoint-based cancer immunotherapy has shown significant efficacy in various cancers, resistance still limits its therapeutic effects. Ubiquitination modification is a mechanism that adds different types of ubiquitin chains to proteins, mediating protein degradation or altering their function, thereby affecting cellular signal transduction. Increasing evidence suggests that ubiquitination modification plays a crucial role in regulating the mechanisms of resistance to cancer immunotherapy. Drugs targeting ubiquitination modification pathways have been shown to inhibit tumor progression or enhance the efficacy of cancer immunotherapy. This review elaborates on the mechanisms by which tumor cells, immune cells, and the tumor microenvironment mediate resistance to cancer immunotherapy and the details of how ubiquitination modification regulates these mechanisms, providing a foundation for enhancing the efficacy of cancer immunotherapy by intervening in ubiquitination modification.

## Introduction

The successful application of immune checkpoint inhibitors and other cancer immunotherapies has brought hope to cancer treatment. However, for certain cancer patients, especially those with metastatic cancer, resistance to tumor immunotherapy makes it challenging for them to benefit from these treatments. Intrinsic factors within cancer cells, such as a lack of antigenic proteins, defects in antigen presentation, and insensitivity to T cell-mediated cytotoxicity, can contribute to the development of resistance to immunotherapy. Extrinsic factors, including inhibitory immune checkpoints, suppressive immune cells, and T-cell functional deficiencies, can also contribute to immunotherapy resistance [1]. To overcome resistance to immunotherapy, numerous novel strategies have been proposed. Enhancing the immunogenicity of tumor cells and shaping a pro-anti-tumor immune microenvironment can be achieved through radiation therapy, chemotherapy, targeted therapy, or the use of oncolytic viruses. Increasing the infiltration of local T cells within the tumor can be accomplished via methods such as cytokines and CAR-T cell therapy. Augmenting the functionality of the immune system involves targeting other inhibitory immune checkpoints, activating immune stimulatory signaling pathways, eliminating immunosuppressive cytokines, activating innate immunity, and enhancing tumor antigen presentation. Methods to overcome resistance to cancer immunotherapy also include inhibiting abnormal gene expression in tumor cells and reducing aberrant signal transduction [2].

The ubiquitination modification process plays a regulatory role in various biological processes in eukaryotic cells, including the cell cycle, gene transcription, and signal transduction. Mechanistically, under the action of ubiquitin-activating enzyme (E1), ubiquitin-conjugating enzyme (E2), and ubiquitin ligase (E3), the lysine residues of target proteins become linked to ubiquitin chains [3]. Ubiquitin molecules can be linked through seven lysine residues, namely K6, K11, K27, K29, K33, K48, and K63, resulting in polyubiquitination. Typically, polyubiquitin chains linked through K48 lead to the degradation of substrate proteins via the ubiquitin-proteasome pathway. On the other hand, polyubiquitin chains linked through K63 participate in various biological functions such as protein activation, intracellular turnover, and protein-protein interactions [4]. Deubiquitinating enzymes (DUBs) can remove ubiquitin chains from proteins, eliminating the ubiquitination signal and rendering ubiquitination modification reversible and controllable [5].

It has been established that ubiquitination modification contributes to the emergence of resistance across diverse cancer therapies. Consequently, interventions targeting the ubiquitin-proteasome pathway, responsible for protein degradation, hold promise for reversing drug resistance in cancer treatment [6]. Ubiquitination modification is involved in the regulation of various immune cell functions and is widely present in the interaction between tumors and immune cells. Therefore, altering the ubiquitination modification pathway has the potential to enhance the efficacy of cancer immunotherapy [7].This review primarily encompasses various resistance mechanisms in cancer immunotherapy and delineates how the ubiquitination modification process regulates these mechanisms within cancer cells. It offers insights into targeting ubiquitination modification to overcome resistance in tumor immunotherapy.

## Tumor-intrinsic factors contribute to resistance to immunotherapy

### Intrinsic resistance of tumor to immunotherapy: WNT/β-catenin signaling pathway

**Fig. 1:**
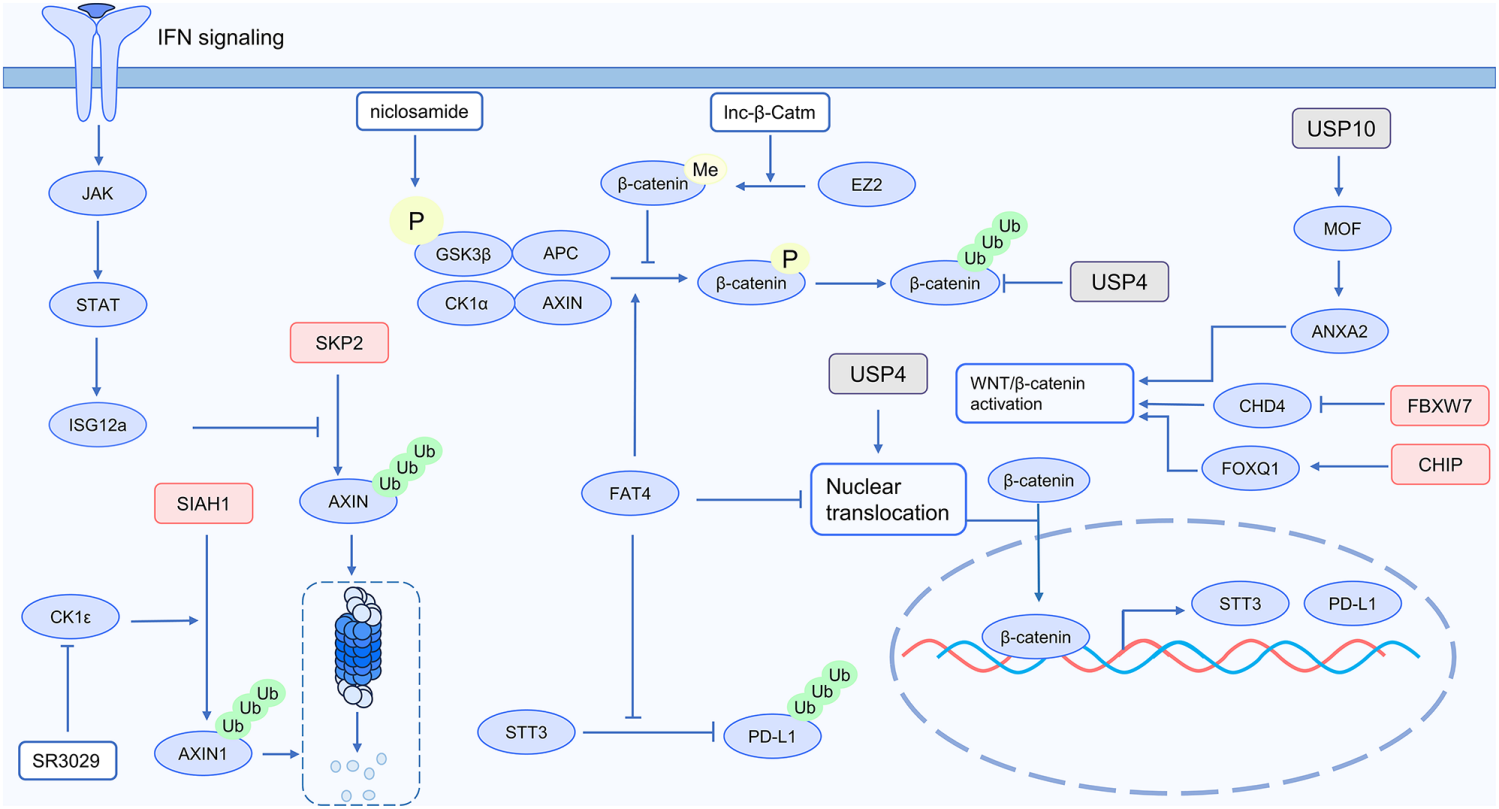
Ubiquitin and WNT/β-catenin signaling in cancer immunotherapy

Through multiple mechanisms, such as inhibiting T cell activation, elevating Indoleamine 2,3-dioxygenase 1 (IDO1) activity, and upregulating PD-L1 levels, the WNT/β-catenin signaling pathway regulates immune checkpoint inhibitor resistance. Immunotherapy can be more effective if the WNT/β-catenin pathway is inhibited [8]. Several studies have demonstrated the role of ubiquitination modification in regulating the WNT/β-catenin pathway. This emphasizes its potential as a target to inhibit tumor immune evasion mediated by WNT/β-catenin and resistance to tumor immunotherapy. The specific mechanism is illustrated in Fig. 1.

The activation of the WNT/β-catenin pathway is regulated by E3 ubiquitin ligases and deubiquitinases. In ovarian cancer (OC), PARP1 stabilizes the protein level of Forkhead box Q1 (FOXQ1) through the E3 ubiquitin ligase Hsc70-interacting protein (CHIP). The WNT/β-catenin pathway is activated by FOXQ1, which accelerates the development of ovarian cancer [9]. In triple-negative breast cancer (TNBC) tissue, chromodomain-helicase-DNA-binding protein 4 (CHD4) promotes the β-catenin nuclear accumulation. The E3 ubiquitin ligase F-box and WD repeat domain-containing 7 (FBXW7) directly binds to CHD4, promoting its ubiquitination and degradation. Tumor development is suppressed and the WNT/β-catenin pathway is greatly inhibited when CHD4 expression is reduced [10]. In esophageal squamous cell carcinoma (ESCC), USP10 deubiquitinates MOF and maintains its stability by interacting with it. Through its histone acetyltransferase activity, MOF facilitates the accumulation of H4K16ac within the ANXA2 promoter. This enrichment, dependent on JUN, stimulates ANXA2 transcription, thereby activating WNT/β-catenin signaling and ultimately stimulating the development of ESCC [11]. In colorectal cancer (CRC) cells, the β-catenin destruction complex component AXIN1 is ubiquitinated and degraded as a result of the interaction between the E3 ubiquitin ligase SIAH1 and AXIN1, which is facilitated by CK1ε. The CK1δ/ε inhibitor SR3029 targets CK1ε, significantly increasing the levels of AXIN1 protein. This inhibits WNT/β-catenin signaling, thereby suppressing tumor initiation and progression in CRC [12]. As a deubiquitinating enzyme, USP4 promotes the stability and localization of the β-catenin in the nucleus. This procedure increases the activation of the WNT/β-catenin pathway [13]. In summary, the WNT/β-catenin pathway is highly regulated by ubiquitination modification, providing a potential method for inhibiting its signal.

Ubiquitination modification degrades β-catenin, resulting in reduced PD-L1 levels, thus reversing tumor immune evasion. Research indicates that FAT4 (FAT Atypical Cadherin 4) regulates β-catenin ubiquitination levels, leading to increased anti-tumor immunity. Mechanistically, β-catenin promotes PD-L1 and N-glycosyltransferase STT3 transcription within the nucleus. STT3 decreases the ubiquitination and degradation of PD-L1 by increasing its glycosylation. AXIN, GSK3β, CK1α, and APC form a degradation complex for β-catenin, facilitating its degradation. The degradation complex-mediated β-catenin ubiquitination and degradation is significantly amplified by overexpression of FAT4. Furthermore, FAT4 inhibits the nuclear translocation and transcriptional activity of β-catenin by anchoring it to the cell membrane. As a result, elevated FAT4 levels decrease the correlation between STT3 and PD-L1, inhibiting PD-L1 glycosylation while promoting the ubiquitination and degradation of PD-L1 via GSK3β [14]. According to another research, interferon influences the ubiquitination of β-catenin, which in turn controls PD-L1 expression. Interferon (IFN) activates the JAK-STAT pathway, which increases the expression of IFN-stimulated genes (ISGs). The E3 ubiquitin ligase SKP2 regulates the ubiquitination and degradation of Axin. When ISG12a is highly expressed, it promotes the binding of ISG12a to SKP2, inhibiting the interaction between Axin and SKP2 and thereby hindering the degradation of Axin. ISG12a maintains the Axin protein level, stabilizing the β-catenin destruction complex and promoting β-catenin degradation. While ISG12a suppresses the WNT/β-catenin pathway, PD-L1 expression levels decrease as β-catenin promotes PD-L1 transcription [15]. Both studies demonstrate that PD-L1 is downregulated as a result of the suppression of WNT/β-catenin signaling by ubiquitination modification, rendering tumors vulnerable to immune cell-mediated cytotoxicity [14, 15].

By regulating the ubiquitination of β-catenin, non-coding RNAs and small molecules also impact on the WNT/β-catenin signaling pathway. The lnc-β-Catm is a type of long non-coding RNA (lncRNA). It facilitates β-catenin engaging with the methyltransferase EZH2, which then methylates the K49 site on β-catenin. By preventing β-catenin from being phosphorylated and ubiquitinated, this methylation event enhances the stability of β-catenin and initiates the WNT/β-catenin signaling cascade. Notably, the level of ubiquitination of β-catenin is markedly increased when EZH2 is inhibited [16]. The anthelmintic drug niclosamide can prevent tumor immune evasion through its modification of the WNT/β-catenin pathway. Mechanistically, niclosamide treatment prevents the nuclear translocation of β-catenin. Additionally, it increases GSK-3β phosphorylation, which facilitates β-catenin ubiquitination and eventual degradation [17].

In summary, ubiquitination modifies the WNT/β-catenin signaling pathway, which affects anti-tumor immunity. Interestingly, the β-catenin degradation complex might be a potential therapeutic target. Many molecules, such as p21-activated kinase 4 (PAK4) and IDO1, participate in activating the WNT/β-catenin pathway [8]. It remains to be studied whether targeting the ubiquitination degradation of these molecules can modulate the WNT/β-catenin pathway.

### Intrinsic resistance of tumor to immunotherapy: interferon signaling pathway

**Fig. 2:**
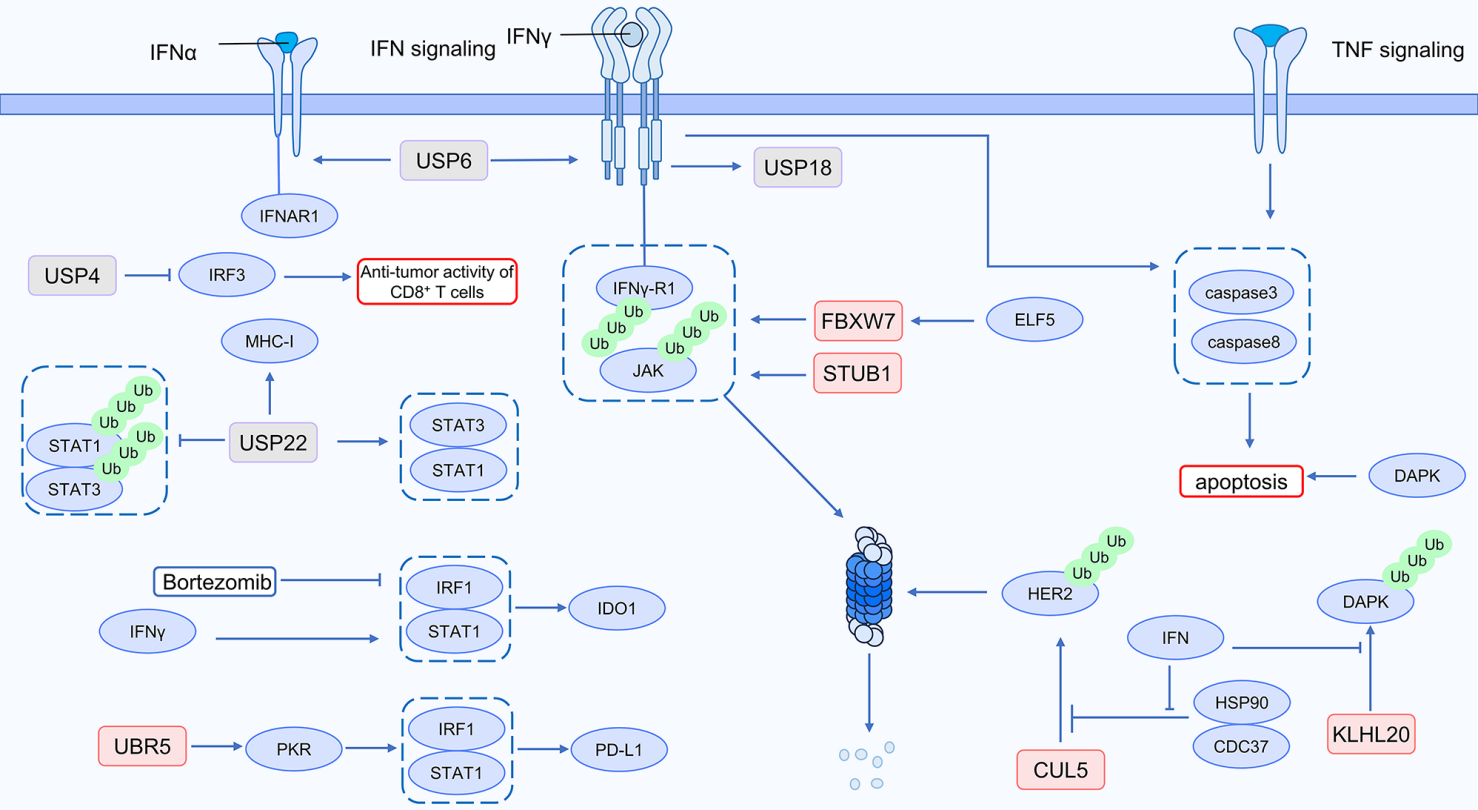
Ubiquitin and IFN signaling in cancer immunotherapy

Interferons increase immune cell maturation and activation, hence mediating antitumor immunity. Tumor cells, however, frequently disrupt interferon signaling pathways, including IFNGR/JAK/STAT, which makes them resistant to interferon stimulation. Furthermore, tumor cells alter interferon signaling to prioritize the production of immunosuppressive molecules like PD-L1 and IDO1 and to increase the expression of ISGs. Consequently, interferons play conflicting roles in tumor progression, posing challenges for their therapeutic involvement in cancer treatment [18]. The mechanisms of resistance to tumor immunotherapy include disruption of interferon signaling transduction. However, the complicated relationship between interferons and the effectiveness of tumor immunotherapy is highlighted by the fact that prolonged activation of interferon signaling in tumor cells can also promote resistance to immunotherapy [19]. Nonetheless, there remains a possibility of successfully improving immunotherapy efficacy by intervening in interferon signaling transduction.The susceptibility of tumor cells to the cytotoxicity of immune cells is controlled via the ubiquitination modifications and interferon signaling pathways together. The specific mechanism is illustrated in Fig. 2.

The signaling pathway of the cytokine IFNγ in tumor cells is influenced by the E3 ubiquitin ligase STUB1. Mechanistically, IFN-γR1 serves as the receptor for IFN-γ, and JAK1 participates in the downstream signal transduction of IFN-γR1. STUB1, through its tetratricopeptide tandem repeat (TPR) domain, targets the K285 residue of IFNγ-R1 and the K249 residue of JAK1, inducing proteasomal degradation of both IFNγ-R1 and JAK1. By promoting IFNγ signal transduction, STUB1 inhibition makes tumor cells more vulnerable to T cells. Furthermore, It increases the efficacy of PD-1 antibodies [20]. SMYD3 promotes immune evasion in human papillomavirus (HPV)-negative head and neck squamous cell carcinoma (HNSCC). Mechanistically, UHRF1 is not only an E3 ubiquitin ligase but also an H3K9me3 reader. UHRF1 recognizes and binds to the promoters of immune-related genes, such as type I interferons (IFNs), which are rich in H3K9me3 methylation. It recruits DNMT1 to CpG DNA methylation sites, thereby repressing the expression of these immune-related genes. This function is independent of its role as an E3 ubiquitin ligase. The reduction of SMYD3 increases the effectiveness of PD-1 antibodies and encourages CD8^+^ T cell migration to tumors [21]. The chemokine CXCL10 recruits CD8^+^ T cells, inhibiting the progression of synovial sarcoma. USP6, by elevating the levels of JAK1, induces CXCL10 through the JAK1-STAT1 pathway, with this inductive effect being more pronounced in the presence of exogenous IFN-α. Additionally, USP6 increases the levels of IFN receptors IFNGR1 and IFNAR1, enhancing cellular sensitivity to IFN [22]. The deubiquitinase USP22 shows a positive correlation with IFN signaling pathway elements in tumor cells, including JAK1, JAK2, STAT1, and IFNGR. It participates in downstream signaling of IFN-γ under the influence of JAK1, promoting the MHC-I complex to be expressed. Low expression of USP22 in tumor cells affects the anti-tumor capability of immune cells. Mechanistically, USP22 deubiquitinates STAT1 and STAT3, preserving their protein stability. The transcription factor STAT is an essential component in the IFN signal [23]. IFN-γ stimulates the expression of USP18 in tumor cells. Increased expression of USP18 in tumor cells increases CD8^+^ T cell activity, and it induces CD4^+^ T cells to produce IL-2 and IFN-γ [24]. Ubiquitination modification also regulates ISG expression. In colorectal cancer (CRC) with a microsatellite stable (MSS) phenotype, USP4 expression is upregulated, impairing the anti-tumor immune response. Mechanistically, USP4 inhibits the K63-linked ubiquitination of TRAF6 and interferon regulatory factor 3 (IRF3), preventing IRF3 nuclear localization and inhibiting IRF3 activation. Knocking down USP4 stimulates the production of ISGs, increases the anti-tumor activity of CD8^+^ T cells, and improves immunotherapy effectiveness [25].

Interferon and ubiquitination modifications collectively impact tumor cell death. Death-associated protein kinase (DAPK) regulates IFN-γ-induced autophagy and apoptosis, modulating the IFN-γ responses of tumor cells. Acting as the E3 ubiquitin ligase for DAPK, the KLHL20–Cul3–ROC1 complex mediates DAPK degradation. Upon exposure to IFN, KLHL20 accumulates within PML nuclear bodies (PML-NB), thus reducing KLHL-mediated ubiquitination of DAPK and enhancing the stability of DAPK [26]. LUBAC is an E3 ubiquitin ligase, with HOIP being its catalytic component. Lower expression of HOIP can lead to increased production of caspase-3 and caspase-8 in tumor cells mediated by TNF and IFN-γ, inducing tumor cell apoptosis. Inhibiting HOIP may increase the cytotoxicity of TNF and IFN-γ in tumor immunotherapy [27].

Interferon and ubiquitination modifications collectively regulate the activation of oncogenic signaling pathways. HER2 breast cancer (BC) cells often exhibit elevated expression of HER2, cell division cycle 37 protein (Cdc37), and Heat shock protein 90 (Hsp90), while the expression of Cullin5 (CUL5) is decreased. CUL5 functions as the E3 ubiquitin ligase for HER2, facilitating its degradation. Hsp90 binds to HER2 with the assistance of the co-chaperone Cdc37, shielding HER2 from degradation by CUL5. Stimulation with IFN-γ can induce the dissociation of Hsp90 from Cdc37 at the post-transcriptional level, reducing their interaction with HER2. This results in elevated levels of CUL5, promoting the proteasomal degradation of HER2, and downregulating signaling pathways associated with tumor growth [28].

However, ubiquitin modifications can also be involved in IFN-γ-mediated immune suppression in cancer. The anti-cancer activities of IFN-γ are widely acknowledged. However, TNBC with heterozygous deletion of the E74-like transcription factor (Elf5) exhibits enhanced IFN-γ signal transduction, consequently, this leads to increased neutrophil infiltration and enhanced metastatic capability of TNBC cells. Mechanistically, ELF5 promotes FBXW7 expression. FBXW7 is an E3 ubiquitin ligase for IFN-γ receptor 1 (IFNGR1), interacting with it and facilitating its degradation. ELF5 deficiency leads to reduced FBXW7 expression, stabilizing the protein levels of IFNGR1 and promoting IFN-γ signal transduction. The increase in tumor cell metastasis resulting from sustained IFN-γ signal transduction can be reduced by blocking PD-L1 [29]. PD-L1 expression can be regulated by UBR5, an E3 ubiquitin ligase, via a non-ubiquitin-dependent approach. The gene Eif2ak2 encodes the RNA-activated protein (PKR), and in the presence of IFN-γ, UBR5 relies on its PABC domain to participate in the transcription of the Eif2ak2 gene, increasing the expression of PKR. This promotes the upregulation of signal transducers and activators of transcription 1 (STAT1) and interferon regulatory factor 1 (IRF1) downstream of PKR. The transcription of PD-L1 is facilitated by the binding of IRF1 and STAT to the PD-L1 promoter [30]. USP15, a deubiquitinase, regulates the generation of T helper 1 (Th1) cells, which generate IFN-γ. Mice lacking USP15 produce excessive IFN-γ, resulting in elevated expression of PD-L1 and CXCL12, creating an immunosuppressive tumor microenvironment. Inhibiting IFN-γ can disrupt the formation of this immunosuppressive microenvironment [31]. Interestingly, a study has suggested that targeting proteasomes in the ubiquitin-proteasome pathway inhibits interferon-mediated tumor immune evasion. IDO expression increases in response to IFN-γ signaling, contributing to cancer immune tolerance. Bortezomib inhibits this IFN-γ-induced IDO expression. Mechanistically, the expression of IDO is controlled by the activation of ISRE and GAS, which necessitate the participation of STAT1 and IRF-1. Upon IFN-γ stimulation, STAT1 is activated, consequently facilitating the upregulation of IDO1 expression. Bortezomib is a proteasome inhibitor that prevents the nuclear translocation and phosphorylation of STAT1, which prevents STAT1-dependent activation of GAS and ISRE. Furthermore, by blocking the NF-κB signaling pathway, Bortezomib reduces the expression of IRF-1 [32].

In summary, targeting the interferon pathway to enhance anti-tumor immunity seems challenging due to the contradictory role of interferons in tumor immunity. However, given the precise control of protein degradation by ubiquitination, identifying key components promoting tumor immune evasion within the interferon pathway, as well as products of interferon-stimulated gene expression, and aiming to degrade them through the ubiquitin-proteasome pathway may represent potential strategies to overcome interferon-mediated resistance to tumor immunotherapy.

### Intrinsic resistance of tumor to immunotherapy: ubiquitin modification and cell death

**Fig. 3:**
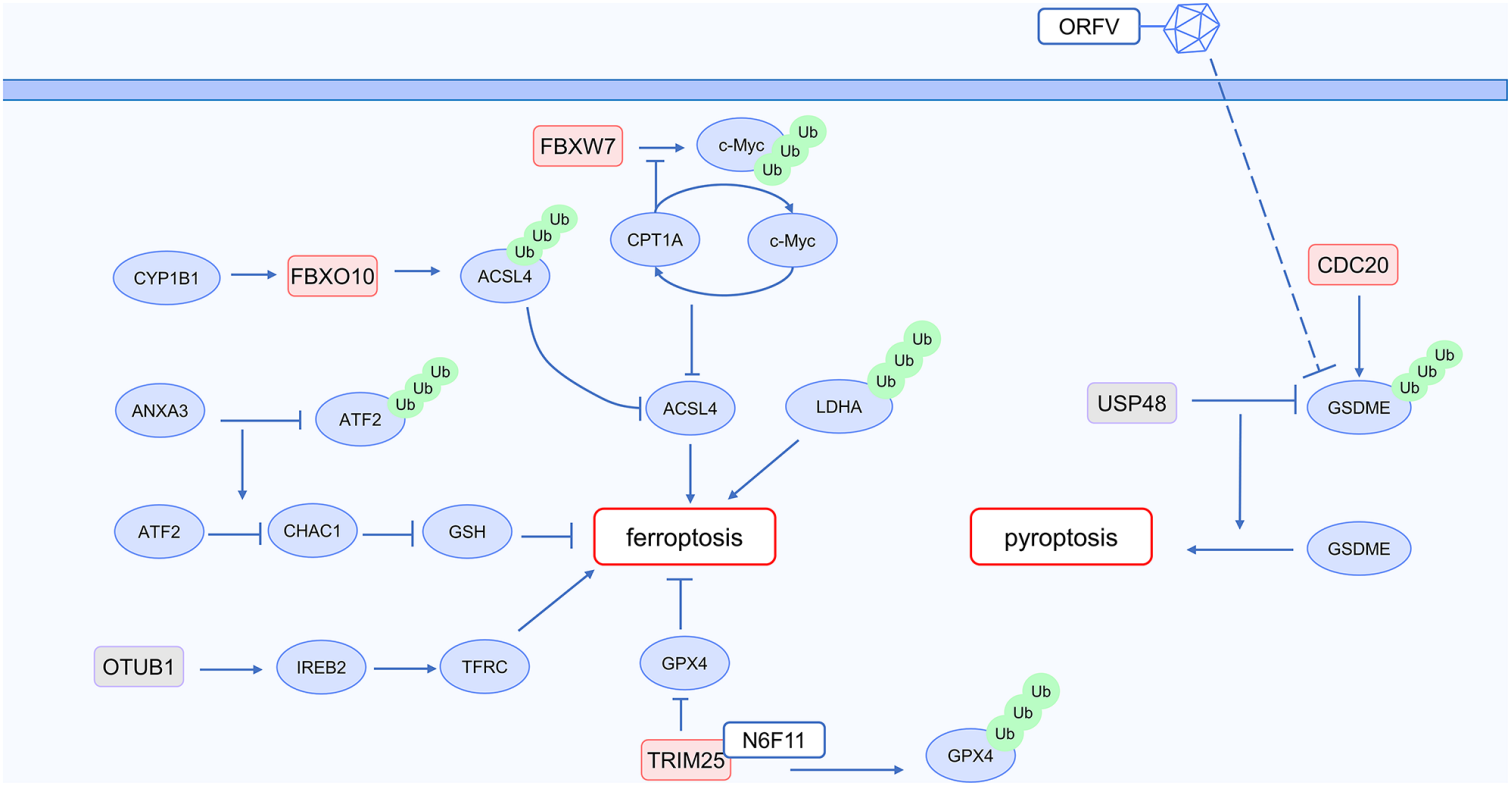
Ubiquitin and cell death in cancer immunotherapy

Ferroptosis induces immunogenic cell death in tumor cells, stimulating the adaptive immune system to enhance anti-tumor activity. It disrupts the immunosuppressive microenvironment of Tregs, reversing immune therapy resistance. Additionally, ferroptosis inhibits the survival of M2 TAMs without affecting M1 TAMs, promoting their reprogramming to increase anti-tumor efficacy. Previous studies have reported that inducing ferroptosis reverses resistance to tumor immunotherapy [33]. In regulating ferroptosis, ubiquitination also plays a significant role. The specific mechanism is illustrated in Fig. 3.

Multiple studies have shown that tumor-associated macrophages inhibit ferroptosis in tumor cells, and ubiquitination modification participates in this process. Tumor-associated macrophages (TAM) produce exosomes rich in annexin A3 (ANXA3), acting on laryngeal squamous cell carcinoma (LSCC) cells and inhibiting their ferroptosis. Mechanistically, ANXA3 inhibits the ubiquitination of ATF2, stabilizing its protein level. ATF2 binds to the promoter region of ChaC glutathione-specific γ-glutamyl cyclotransferase 1 (CHAC1), exerting suppressive control over its expression, thus downregulating CHAC1-mediated degradation of GSH and attenuating ferroptosis [34]. M2 TAMs suppress ferroptosis in lung cancer stem cells (LCSCs). Mechanistically, in LCSCs, Carnitine palmitoyl transferase 1 A (CPT1A) competitively binds to c-Myc, preventing the ubiquitination and degradation of c-Myc mediated by FBXW7, thereby maintaining the protein levels of c-Myc. Additionally, c-Myc enhances the transcription of CPT1A, forming a positive feedback loop wherein CPT1A and c-Myc mutually reinforce the expression and activity of each other. This positive feedback loop activates the NRF2/GPX4 antioxidant system while downregulating Acyl-CoA synthetase long-chain family member 4 (ACSL4) to reduce the production of phospholipid polyunsaturated fatty acids (PUFA-PLs), thereby inhibiting ferroptosis. M2 macrophages increase the secretion of L-carnitine in a BBOX1-dependent manner. Within tumor cells, L-carnitine penetrates and initiates the activation of the positive feedback loop involving CPT1A/c-Myc, thereby inhibiting ferroptosis in LCSCs [35].

Ubiquitination modification regulates multiple molecules involved in the process of ferroptosis. ACSL4 facilitates the transformation of arachidonic acid (AA) into arachidonyl-CoA, which encourages cellular ferroptosis. Conversely, cytochrome P450 1B1 (CYP1B1) reduces the protein levels of ACSL4, thereby enhancing cellular resistance to ferroptosis and diminishing the effectiveness of immune checkpoint inhibitors. Mechanistically, CYP1B1 participates in AA metabolism, converting it into 12-HETE and 20-HETE. The PKC signaling pathway is activated by 20-HETE, leading to elevated expression of the E3 ubiquitin ligase FBXO10. Consequently, FBXO10 facilitates the ubiquitination and degradation of ACSL4 [36]. Inhibiting ferroptosis induced by RSL3 is achieved through the overexpression of L-lactate dehydrogenase A (LDHA). Moreover, apolipoprotein L3 (APOL3) interacts with LDHA, facilitating its ubiquitination and subsequent proteasomal degradation. The APOL3-LDHA axis increases IFNγ levels in colorectal cancer cells, reduces lactate concentration, promotes tumor ferroptosis, and enhances the efficacy of anti-PD-1 immunotherapy [37]. The deubiquitinase OTUB1 is expressed in intestinal epithelial cells and goblet cells. OTUB1 deubiquitinates iron-responsive element-binding protein 2 (IREB2), maintaining its protein level stability. IREB2 serves as an intracellular iron sensor, promoting the stability of transferrin receptor protein 1 (TFRC) mRNA and facilitating cellular iron uptake. Tumor cells with high OTUB1 expression exhibit a significant increase in cellular iron content, leading to oxidative stress, promoting ferroptosis, and stimulating the release of damage-associated molecular patterns (DAMPs), subsequently initiating immunogenic cell death, reinforcing anti-tumor immune responses [38]. The small molecule N6F11 serves as an inducer of ferroptosis, causing degradation of glutathione peroxidase 4 (GPX4) in tumor cells without affecting GPX4 levels in immune cells. Mechanistically, GPX4 acts as an inhibitor of ferroptosis. Binding to the E3 ubiquitin ligase TRIM25, N6F11 facilitates the K48-linked ubiquitination and subsequent degradation of GPX4 by TRIM25. This induces ferroptosis in cancer cells. N6F11 also enhances the effectiveness of immunotherapy targeting PD-L1 [39].

The above studies indicate that crucial molecules implicated in the mechanism of ferroptosis, such as ACSL4 and GPX4, are regulated by ubiquitination. Further research could focus on whether ubiquitination can also target other key molecules in the ferroptosis process, such as the cystine/glutamate antiporter (xCT) system [33], to enhance methods for regulating ferroptosis and thus explore potential strategies to reverse immune therapy resistance.

Pyroptosis, characterized as an inflammatory mode of cell death, has the potential to increase the infiltration of CD8^+^ T cells within tumors, hence increasing the efficacy of immunotherapy [40]. Ubiquitin modification also plays a role in regulating pyroptosis. Specifically, the deubiquitinase USP48 interacts with gasdermin E (GSDME), eliminating K48-linked ubiquitination at sites K120 and K189, thereby stabilizing its protein levels and facilitating cell pyroptosis. In a murine tumor model, upregulation of USP48 promoted the therapeutic effectiveness of PD-1 inhibitors [41]. Interacting with the 7RNFL10 motif of GSDME via its repeated WD40 domain, the E3 ubiquitin ligase CDC20 induces the ubiquitination and subsequent degradation of GSDME. Suppression of CDC20 expression results in elevated levels of GSDME, thereby promoting pyroptosis and enhancing anti-tumor immune responses. The combination of CDC20 inhibitors with anti-PD-1 immunotherapy demonstrates a more significant therapeutic effect [42]. Oncolytic viruses also induce pyroptosis. The oncolytic Parapoxvirus ovis (ORFV) reduces the ubiquitination of GSDME, stabilizing it and initiating cell pyroptosis, thereby enhancing tumor sensitivity to immune checkpoint inhibitors [43]. In the nucleus, USP18 regulates immunogenic cell death in a manner that is independent of its deubiquitinase activity. After IFN treatment, the loss of USP18 not only induces pyroptosis through GSDMD but also, with the involvement of PLK2, triggers pyroptosis through GSDME, leading to suppressed tumor growth in USP18-depleted conditions [44].

In summary, targeting ubiquitination in the pyroptosis pathway stabilizes the protein levels of key components in this pathway, offering a potential approach to reverse resistance to cancer immunotherapy.

## Immune cells are involved in regulating resistance to immunotherapy

### Immune cells regulate the effect of immunotherapy: through chemokines

Chemokines are involved in the processes of resistance to immunotherapy by influencing the infiltration and activity of different immune cells inside the tumor microenvironment [45]. Eliminating chemokine-induced negative effects on anti-tumor immunity helps to overcome immunotherapy resistance.

Ubiquitination modification and chemokines collaborate to regulate immune cell recruitment and activation, increasing antitumor immunity. In colorectal cancer, immune evasion is related to Tribbles homolog 3 (TRIB3). Overexpression of the chemokine C-X-C motif chemokine ligand 10 (CXCL10) eliminates the immunosuppressive effect of TRIB3 on CD8^+^ T cells. Mechanistically, The acetylation of the TRIB3 K240 site is catalyzed by P300, which reduces the interaction of TRIB3 with the E3 ubiquitin ligase SIAH1, preventing its degradation. The migration of CD8^+^ T lymphocytes is mediated by CXCL10, and STAT1 is the transcription factor involved in CXCL10 transcription. TRIB3 activates the EGFR-STAT3 signaling pathway to inhibit STAT1 transcription, thereby suppressing CXCL10 expression [46]. T cell activation induced by TCR stimulation and cytokines like IL-2 and IL-15 is amplified when Cytokine-Inducible SH2-containing Protein (CISH) is lost in T cells. Increased FBXO38 levels in activated T cells cause PD-1 to be ubiquitinated and degraded. CAR-T cells with CISH knockout also demonstrate increased anti-tumor capabilities [47]. T cell-specific cytokines are induced to express via the transcription factor NFATc2. By stabilizing the E3 ligase MDM2, deubiquitinase USP15 inhibits T cell activation by targeting NFATc2 for degradation [48].

Cytokines stimulate immune cell activity through the NF-κB signaling pathway, and ubiquitination modification regulates this process. The cancer immune suppression process is mediated by the E3 ubiquitin ligase BIRC2. In cancer cells, BIRC2 knockdown upregulates CXCL9 expression and non-canonical NF-κB signaling. CXCL9 contributes to the recruitment of NK and T cells, which inhibits the proliferation of tumors. In breast cancer, BIRC2 knockdown increases the effectiveness of PD-1 blockade [49]. Belonging to the NF-κB family, p50, when elevated, stimulates the production of pro-inflammatory chemokines such as CCL3, CCL4, and CCL5. Consequently, this process leads to the recruitment of NK cells and macrophages, contributing to a tumor-suppressive effect. The ubiquitin ligase KPC1 ubiquitinates p105, leading to its processing in the proteasome to generate p50 [50].

Ubiquitin-mediated regulation of chemokine signaling also leads to the establishment of an immunosuppressive microenvironment. Metabolic reprogramming in T cells, such as increased expression of glycolysis-related enzymes, is the manifestation of activated T cells, and AKT is a key kinase mediating T cell metabolic reprogramming. Downregulation of the deubiquitinase Otub1 significantly promotes tumor infiltration by T cells, enhancing anti-tumor immunity. Mechanistically, IL-15 strongly induces the interaction between Otub1 and AKT, inhibiting the K63-linked ubiquitination of AKT. This leads to a conformational change in AKT, inhibiting its interaction with membrane lipid PIP3 through its pleckstrin homology (PH) domain. As a result, AKT activation becomes challenging, suppressing T cell functionality [51]. Ubiquitination modification of cytokine signaling pathways not only affects T cell function but also mediates immune suppression by influencing macrophage function. Through inhibiting the expression of CCL2, the SLAMF7 protein achieves the reprogramming of macrophage polarization. Low SLAMF7 expression promotes M2-like polarization of macrophages. Mechanistically, ATF2-mediated CCL2 transcription is facilitated by JNK and p38, two members of the MAPK signaling cascade. SLAMF7 binds to the SH2 domain-containing adaptor protein B (SHB), which recruits SH2 domain-containing inositol phosphatase 1 (SHIP1) to inhibit the K63-linked ubiquitination of TRAF6. This inhibition suppresses the activation of the p38 and JNK pathways mediated by TRAF6, leading to reduced CCL2 expression. In tumor cells with low SLAMF7 expression, using a CCR2 antagonist to inhibit the CCL2/CCR2 axis, generates a greater anti-tumor impact when combined with PD-1 antibody [52]. E3 ligase UBR5 promotes the release of paracrine factors like CCL2 and CSF-1, leading to increased TAM infiltration and promoting tumor cell proliferation [53].

Cytokines also limit the activity of immune cells through the NF-κB signaling pathway, and ubiquitination modification regulates this process. USP10 deubiquitinates and stabilizes NLRP7. Activated NLRP7 stimulates the NF-κB signaling pathway, promoting the secretion of CCL2, inducing M2-like TAM polarization, and fostering an immune-suppressive microenvironment [54]. PPPM1B serves as an inhibitor of NF-κB, and the deubiquitinating enzyme USP12 interacts with PPPM1B, maintaining the stability of PPPM1B protein levels. The expression of USP12 reduces cytokine levels such as CXCL1 and CCL2, which are associated with NF-κB activation. Inhibiting USP12 leads to the promotion of these cytokines, contributing to the establishment of an immunosuppressive microenvironment [55].

**Fig. 4:**
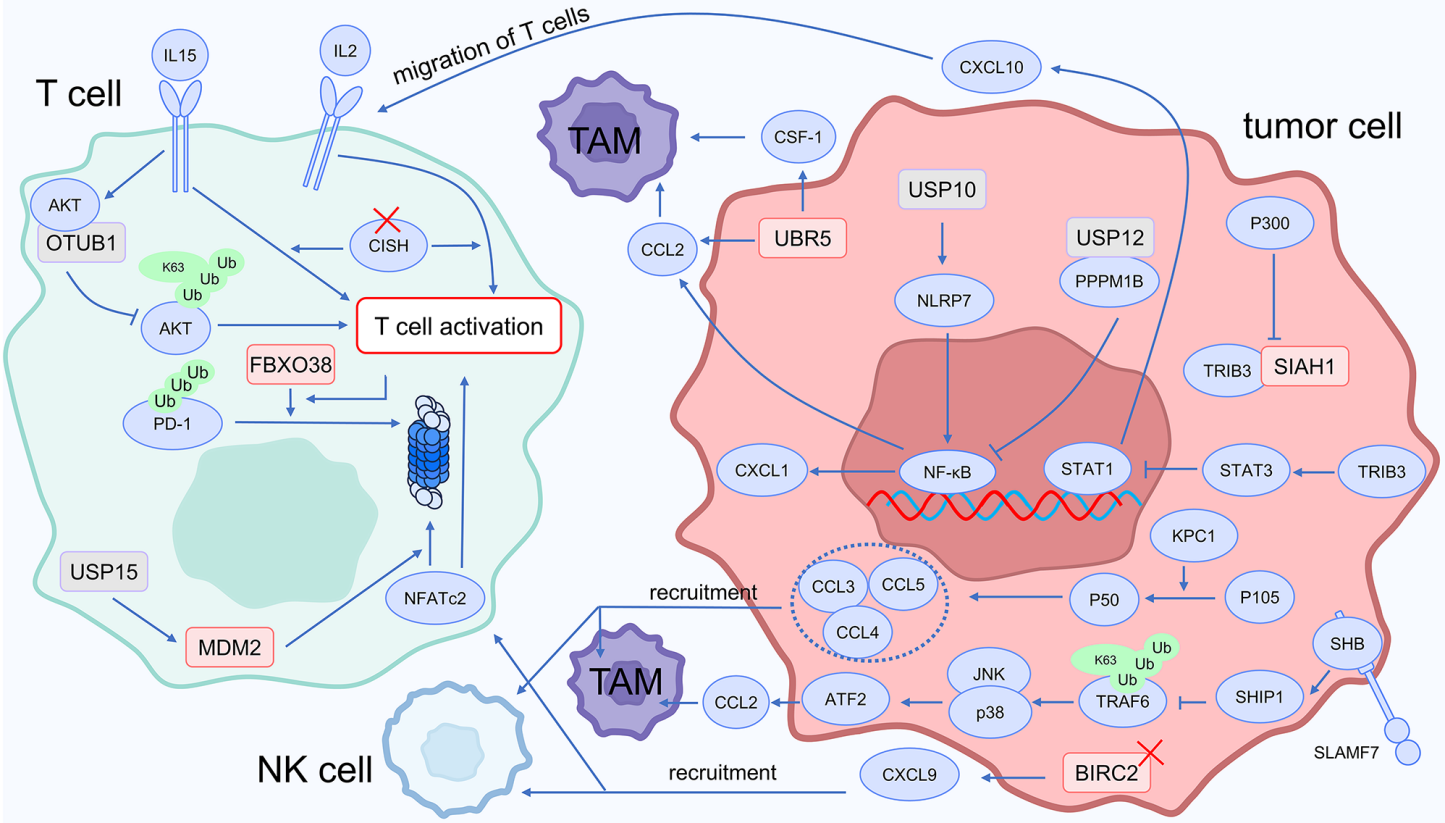
Ubiquitin and chemokines in cancer immunotherapy

The mechanisms mentioned above are illustrated in Fig. 4. In conclusion, implementing ubiquitination modifications to enhance cytokine anti-tumor effects while suppressing pathways that promote carcinogenic cytokine production offers an intriguing strategy for overcoming cancer immunotherapy resistance. Remarkably, the NF-κB pathway shows promise as a target for anti-tumor therapies.

### Immune cells regulate immunotherapy effect: TGF-β signaling pathway affects immune cell function

**Fig. 5:**
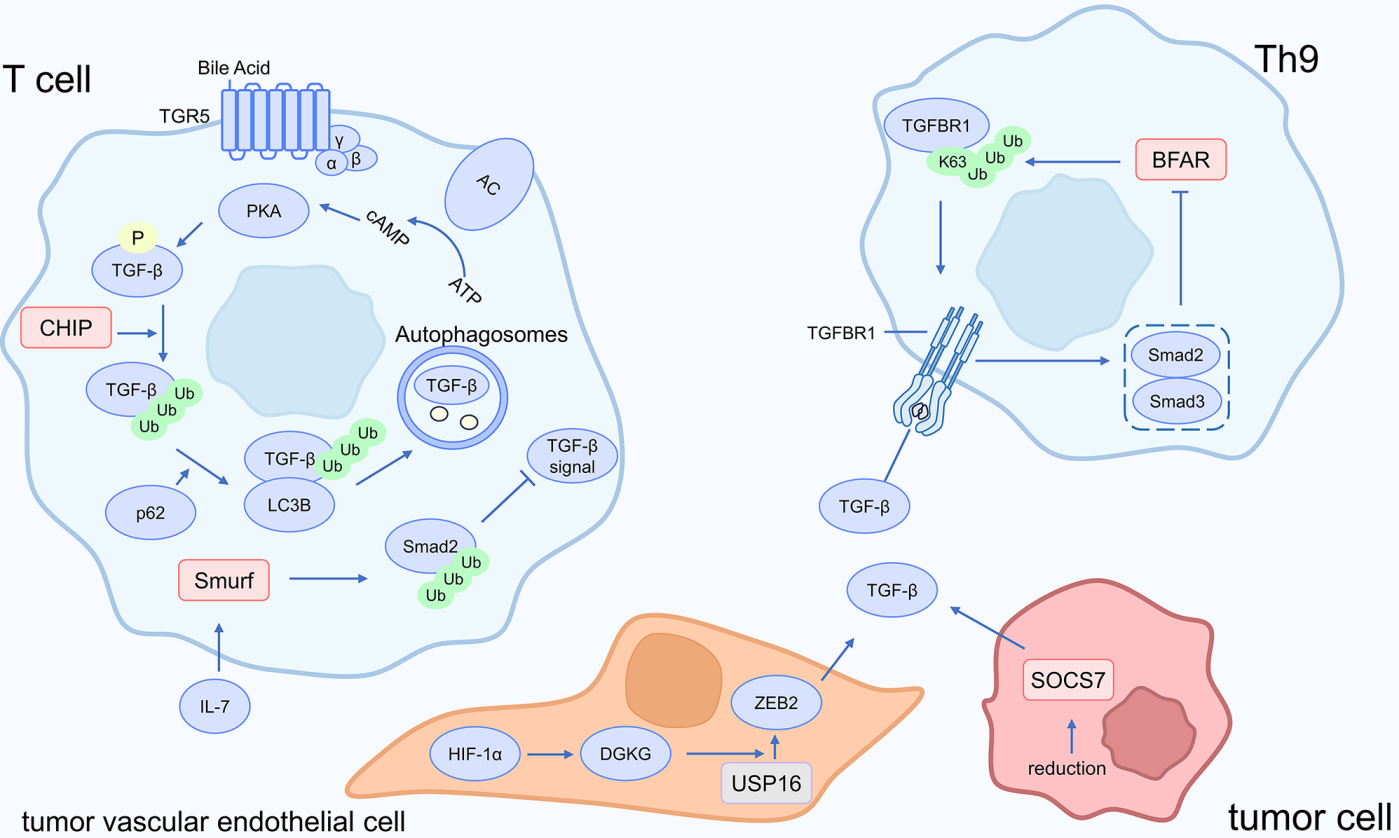
Ubiquitin and TGF-β signaling in cancer immunotherapy

TGF-β suppresses the anti-tumor immunity generated by T cells by influencing their migration, differentiation, and proliferation. Inhibiting TGF-β signaling enhances T cell anti-tumor responses [56]. TGF-β promotes resistance to immunotherapy, while TGF-β inhibitors enhance the efficacy of immunotherapy [1]. Ubiquitination modification participates in the regulation of TGF-β signaling transduction. The specific mechanism is illustrated in Fig. 5.

In tumor vascular endothelial cells (ECs), under hypoxic conditions, HIF-1α activates the transcription of diacylglycerol kinase gamma (DGKG). DGKG recruits USP16 to deubiquitinate ZEB2, maintaining its stability, resulting in increased secretion of TGF-β1, driving the differentiation of Tregs, and promoting tumor immune evasion. Targeting DGKG improves the therapeutic effectiveness of PD-1 antibodies [57]. IL-7 stimulates T cells to upregulate the ubiquitin ligase Smurf, which targets Smad2 for degradation and inhibits the TGF-β signaling pathway. IL-7 functions as an adjuvant and can increase the effects of various immunotherapies, such as tumor vaccinations [58]. CBL-B-deficient T cells resist immunological suppression from TGF-β and Tregs, leading to an enhanced anti-tumor immune response [59]. TGF-β and other immune-suppressive factors are expressed when the E3 ubiquitin ligase SOCS-7 is underexpressed. However, the specific mechanism remains unclear [60]. A secondary bile acid (BA) called ursodeoxycholic acid (UDCA) is essential for inhibiting Treg activation, enhancing anti-tumor immune responses, and degrading TGF-β. Mechanically, Bile acids (BAs) stimulate the membrane receptor TGR5. Upon activation by UDCA, TGR5 initiates a cascade, allowing adenylyl cyclase to become active, cAMP levels to rise, and subsequent activation of protein kinase A (PKA). PKA phosphorylates TGF-β, facilitating E3 ligase CHIP-mediated ubiquitination of TGF-β. The ubiquitinated TGF-β then interacts with p62 through the ubiquitin-associated (UBA) domain of p62. p62 functions as an adapter protein that stimulates TGF-β and LC3B interaction, directing TGF-β to autophagosomes and promoting autophagy-related degradation of TGF-β [61].

However, TGF-β does not only exert immunosuppressive effects. Th9 cells possess potent anti-tumor activity, and TGF-β is the primary cytokine inducing the generation of Th9 cells. The E3 ubiquitin ligase bifunctional apoptosis regulator (BFAR) activates TGF-β signaling by K63-linked ubiquitination at the K268 site of TGF-βR1. Prolonged stimulation by TGF-β, however, activates SMAD2/3, which inhibits BFAR transcription, forming a negative feedback regulatory loop [62]. In conclusion, ubiquitination of TGF-β may improve T cell antitumor efficacy and reverse immunotherapy resistance. More study is needed to discover whether activating Th9 through TGF-β has substantial advantages against cancer.

### Immune cells regulate immunotherapy effect: T cell dysfunction and abnormal TCR signal transduction

**Fig. 6:**
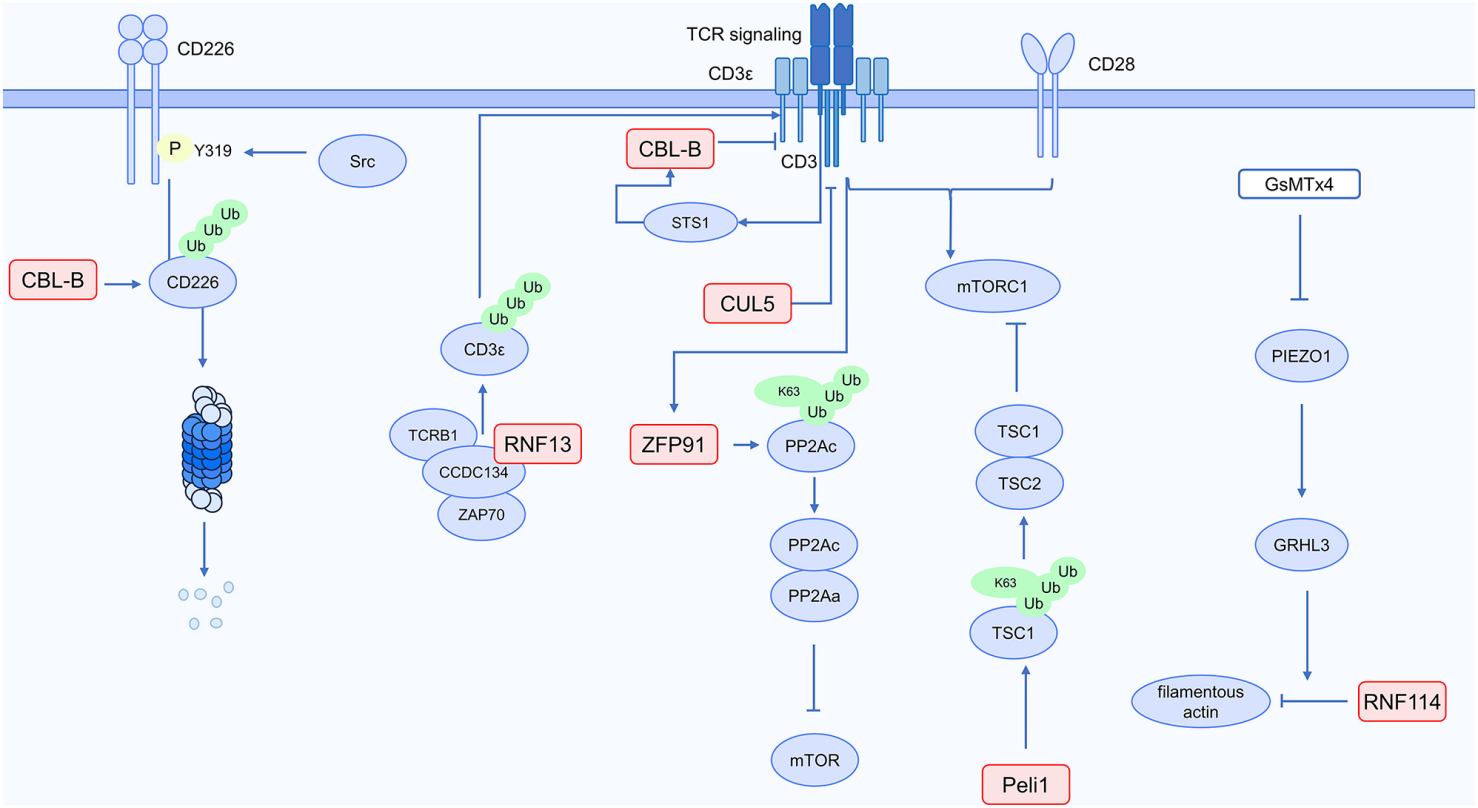
Ubiquitin and functions of T cells in cancer immunotherapy

The effectiveness of cancer immunotherapy is impaired by T cell exhaustion and dysfunction as well as by the high expression of PD-1 [63]. T cell function is regulated by ubiquitination modification. The specific mechanism is illustrated in Fig. 6. It is interesting to note that the E3 ligase CBL-B seems to be essential to this process.

Exhausted T cells exhibit a decline in their cytotoxicity against tumor cells, accompanied by downregulation of IFN-γ and TNF-α expression. The expression of inhibitory receptors PD1, Tim, and LAG3 increases concurrently. Higher levels of the CBL-B have been identified in tumor-infiltrating lymphocytes (TILs) that are exhausted and positive for Tim3 and PD1. The production of granzyme B, IFN-γ, TNF-α, and IL-2 is restored in these TILs when CBL-B is deleted. As a result, CD8^+^ T cell exhaustion is greatly influenced by CBL-B [64]. When CD226 on CD8^+^ T cells attaches to CD155 on tumor cells, CD226 is degraded. CD226 deficiency causes the generation of inhibitory receptors, resulting in T-cell dysfunction. The mutation of the Y319 site in CD226 increases the expression of CD226 and demonstrates potent anti-tumor immune capability. Mechanistically, CD155 facilitates the internalization of CD226 into cells, a process associated with Src kinase-induced phosphorylation of CD226 at the Y319 site. Internalized CD226 undergoes ubiquitination and proteasomal degradation under the action of CBL-B [65]. To summarize, a possible strategy to prevent T cell exhaustion and improve the effectiveness of immunotherapy is to target CBL-B in T cells.

Cancer immunotherapy resistance may be mediated by a deficiency of T cells, particularly those with tumor antigen-specific T-cell receptors (TCRs) [1]. This implies that abnormalities in TCR signaling could affect how effectively cancer immunotherapy performs. TCR signal transduction can be impacted by ubiquitination modification, which may affect T cell function and immunotherapy results. The E3 ubiquitin ligase CUL5 in CD8^+^ T cells suppresses T cell function by adversely regulating the TCR and IL2 pathways. T cell anti-tumor activity is increased when CUL5 is deleted in CD8^+^ T cells, leading to an elevation of JAK/STAT and TCR signaling. Consequently, targeting CUL5 could be an effective approach to elevate the efficacy of immunotherapy [66]. T cells with low CCDC134 (coiled-coil domain containing 134) expression exhibit impaired differentiation and insensitivity to stimulation of the TCR signal. Mechanistically, T cell activation is regulated by the ubiquitination of the TCR-CD3 complex. CCDC134 not only interacts with proteins such as TCRB1 and Zap70 that regulate the synthesis and turnover of the TCR-CD3 complex but also interacts with proteins such as E3 ubiquitin ligase RNF13 that mediate ubiquitination. CCDC134 promotes polyubiquitination of CD3ε in the TCR-CD3 complex at K29, K33, and K48 linkages. The ubiquitin chains formed at K29 and K33 can inhibit the proteasomal degradation of proteins, leading to an increase in surface TCR levels. Defective CCDC134 decreases T-cell activation and facilitates TCR downregulation by preventing CD3ε from accumulating and blocking the formation of the TCR-CD3ε complex [67].

CBL-B is closely associated with TCR signal transduction. T-cell activity is suppressed due to the acidic microenvironment. Mechanistically, within T cells, STS1 relies on its phosphatase domain to sense acidic conditions, with a decrease in pH enhancing the catalytic efficiency of STS1. The Y709 site of E3 ubiquitin ligase CBL-B is phosphorylated by ZAP70, recruiting STS1 upon stimulation of the TCR signal. STS1 binds to the PPVPP motif of CBL-B via its SH3 domain, and in an acidic environment, this interaction further inhibits TCR signal transduction. In the acidic tumor microenvironment, deletion of either STS1 or CBL-B increases T cell anti-tumor activity [68]. Another study showed that the upregulation of Cbl-b by CD8^+^ T cells is encouraged by the binding of PD-1 on CD8^+^ T cells to PD-L1 on dendritic cells (DCs). TCR signal transduction is inhibited by the downregulation of TCR on CD8^+^ T cells as a result of increased Cbl-b expression. CD8^+^ T cells and DCs-mediated anti-tumor immunity can be enhanced by blocking the PD-L1/PD-1 interaction [69].

However, more research is needed to determine whether increasing TCR signal transduction could elevate T-cell activation and increase their anti-tumor potential. An essential part of the intracellular signal transduction process of TCR activation is performed by the linker for activation of T cells (LAT). T-cell signal transduction is weakened as a result of LAT ubiquitination. The LAT mutant 2KR, where lysine residues are replaced with arginine, resists ubiquitination modification and enhances T-cell signal transduction. However, the expression of 2KR LAT does not exhibit a clear impact on tumor clearance. Therefore, the augmentation of T-cell activity in vitro does not necessarily translate to increased efficacy in vivo, highlighting the need for further research into the in vivo impact of 2KR LAT on anti-tumor immunity [70].

Under stimulation from the TCR signaling pathway, ubiquitination modification also influences T cell metabolism, thereby impacting T cell function. Metabolic reprogramming in T cells is induced by stimulation via TCR/CD28 and growth factor signals, with mTORC1 kinase implicated. T cell differentiation and function execution are facilitated by this metabolic reprogramming. By promoting the K63-linked ubiquitination of TSC1, the E3 ubiquitin ligase Peli1 assists in the connection between TSC1 and TSC2, maintaining TSC2 levels, and establishing the TSC1-TSC2 complex, which inhibits mTORC1 activity. A deficiency in Peli1 causes T cells to activate mTORC1, which is reflected in increased glycolysis and other metabolic processes. The anti-tumor potential of T lymphocytes is increased by the deletion of Peli1 [71]. T-cell glycolysis is inhibited by the E3 ubiquitin ligase ZFP91. Mechanistically, the serine/threonine protein phosphatase 2 A (PP2A) suppresses T cell glycolysis mediated by the mTORC1 signaling pathway, thereby inhibiting T cell anti-tumor immunity. Stimulation of the TCR signal leads to cytoplasmic translocation of ZFP91. ZFP91 facilitates PP2A complex formation to sustain the phosphorylation activity of PP2A by encouraging K63-linked ubiquitination of PP2Ac, enhancing the connection between PP2Ac and PP2Aa. Knockout of ZFP91 enhances T cell anti-tumor capability [72].

Ubiquitination modification also affects the mechanical characteristics of T lymphocytes, which is related to their antitumor potential. PIEZO1 serves as a mechanical sensor in T cells, and T cell antitumor capabilities are increased and their traction forces are improved by inhibiting PIEZO1. Mechanistically, RNF114, an E3 ubiquitin ligase, is expressed in CD8^+^ T cells as a consequence of PIEZO1 promoting transcription factor GRHL3 expression. RNF114 interacts with filamentous actin to lower its levels, which in turn reduces T cell traction forces. GsMTx4 is an inhibitor of PIEZO1 that exhibits anti-tumor effects in mice. Combining GsMTx4 with PD-1 antibodies shows even stronger anti-tumor effects [73].

In summary, ubiquitination modification affects T cell antitumor activity by regulating TCR signaling transduction, T cell metabolism, and various other mechanisms. Targeting ubiquitination modification of critical components in T cell activation pathways could assist in overcoming immunotherapy resistance.

### Immune cells regulate immunotherapy effect: DC cells

Dendritic cells (DCs) are essential for the presentation of tumor antigens, T cell recruitment, and the enhancement of cytotoxic T cell activity. They additionally contribute to overcoming resistance to cancer immunotherapy and decreasing tumor immune evasion. Nevertheless, the antitumor effects that DCs mediate can be impaired by elements like PD-1 expression on DCs and reduced expression of chemokines that attract DCs [74]. A prospective approach to overcome immunotherapy resistance is to increase the anti-tumor effectiveness of dendritic cells (DCs).

PD-L1 regulates the activity of DCs, and this mechanism is related to ubiquitination modification. Casein kinase 2 (CK2) phosphorylates PD-L1 in DCs at Th285 and Th290 sites, breaking the binding of PD-L1 to the CUL3 E3 ligase. The PD-L1 protein is stabilized by this phosphorylation, which additionally inhibits DC function. By inhibiting CK2, PD-L1 levels in DCs can be decreased, which restores their ability to activate T cells [75]. BAP1 possesses deubiquitinase activity, which inhibits protein degradation. C-C chemokine receptor 5 (CCR5) is highly expressed in clear cell renal carcinoma with BAP1 mutations, forming an immunosuppressive microenvironment. By inhibiting CCR5, antigen presentation by DCs is enhanced, PD-L1 expression in cancer is decreased, and the immunosuppressive microenvironment is reversed [76].

The migratory capacity of DCs is also influenced by ubiquitination modification. Transgelin-2 is a small molecular protein that promotes actin rearrangement in mature DCs, thereby facilitating DC migration. Natural Transgelin‐2 can be ubiquitinated and degraded. Substituting lysine (K) 78, the ubiquitination site of Transgelin‐2, with arginine (R), and increasing the recombinant Transgelin‐2 protein stability within DCs by incorporating a protein transduction domain (PTD), significantly enhances DC contact with T cells, promoting anti-tumor immunity [77].

The function of DCs is influenced by the amount of MHC-II on them, which is regulated by ubiquitination modification. Peptide-MHC II complexes (pMHC-II) are ubiquitinated in early endosomes by the E3 ubiquitin ligase March-I, which causes pMHC-II to be degraded by lysosomes. However, MHC-II molecules without antigen peptides remain stable. This selectively ubiquitination-mediated turnover of pMHC-II molecules contributes to increasing the MHC-II molecules diversity of DCs, hence raising the activity of DCs [78]. Reduced destruction of MHC II from the cell surface, which is accomplished by ubiquitination, is linked to the maturation process of DCs. When compared to immature DCs, mature DCs have lower MHC II ubiquitination levels and a reduced rate of lysosomal breakdown, which causes MHC II to accumulate in the DCs [79]. Further research should focus on the relationship between the MHC-II level of DCs and the anti-tumor capacity of DCs, even though the MHC-II level has been demonstrated to be associated with DC function.

**Fig. 7:**
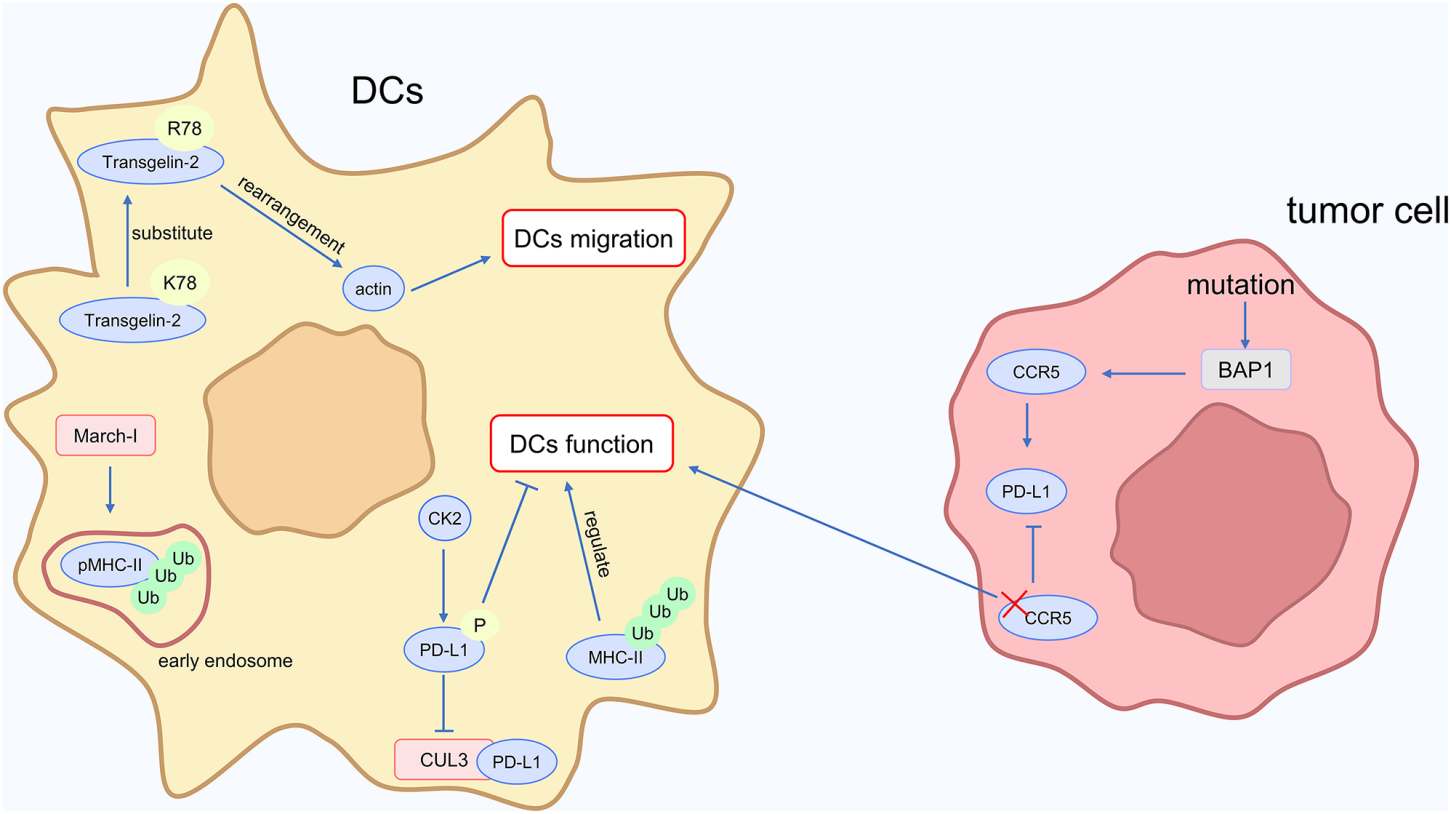
Ubiquitin and DC cells in cancer immunotherapy

The mechanism by which ubiquitin modification regulates dendritic cell (DC) function, as described above, is illustrated in Fig. 7. To sum up, ubiquitination modification regulates the function of DCs. Further investigation could concentrate on two aspects: first, exploring the metabolism of DCs, including the influence of lactate on DC function; second, examining the regulation of DC function by immune checkpoint molecules such as TIM-3. This research direction may reveal whether promoting ubiquitination modification in these processes could enhance the anti-tumor properties of DCs.

### Immune cells regulate immunotherapy effect: NK cells

Improving natural killer (NK) cell function could contribute to overcoming resistance to tumor immunotherapy [2]. Ubiquitin modification regulates the functioning of NK cells.

The E3 ligase CBL-B restricts the cytotoxicity of NK cells against tumors. NK cells deficient in CBL-B exhibit more significant cytotoxicity. Importantly, the lack of CBL-B has no impact on NK cell development, maturation, or in vivo dispersion, suggesting a novel strategy for immunotherapy [80]. According to another study, NK cells derived from mice lacking CBL-B have strong anti-tumor immune activity and are resistant to immune suppression caused by PD-1/PD-L1 [59]. By modifying TAM kinases, CBL-B affects the antitumor activity of NK cells. Mechanistically, the TAM family of cell-surface tyrosine kinase receptors, upon binding with their ligand GAS6, induces the ubiquitination of all components of TAM—Tyro3, Axl, and Mer—by CBL-B. As a result, TAM receptors are downregulated, and NKG2D-activated NK cell proliferation is inhibited. NK cells with CBL-B knockout resist this proliferation inhibition. TAM kinase inhibitors and the anticoagulant warfarin may improve NK cell activity [81].

Several E3 ligases, besides CBL-B, function to regulate the cytotoxicity of NK cells. The recruitment and activation of Src homology 2 (SH2) domain-containing protein tyrosine phosphatase-1 (SHP-1) by inhibitory receptors is related to the impaired function of NK cells. Mechanistically, in NK cells, phosphorylated LAT is recognized by the E3 ligases c-Cbl and Cbl-b, which then ubiquitinate and degrade LAT. This enhances inhibitory receptor-mediated suppression of NK cell cytotoxicity. Moreover, SHP-1 dephosphorylates the Tyr132 residue of LAT in NK cells, thereby eliminating the recruitment of phospholipase C-γ1 (PLC-γ1) and PLC-γ2 to the immunological synapse between NK cells and cancer cells. These processes inhibit the killing impact on target cells as well as the degranulation of NK cells. Enhancing NK cell killing capacity can be achieved by inhibiting LAT ubiquitination [82]. The lysis process of target cells is facilitated by the E3 ubiquitin ligase NK lytic-associated molecule (NKLAM) that exists in NK cells. Uridine-cytidine kinase-like-1 (UCKL-1) increases tumor cell survival, while NKLAM ubiquitinates UCKL-1, causing its degradation. Tumor cells that express less UCKL-1 undergo apoptosis as a result, rendering them more sensitive to NK cell cytotoxicity [83].

E3 ubiquitin ligases regulate the migration of NK cells. The capacity of NK cells to infiltrate tumors is related to the Aryl hydrocarbon receptor (AHR). Mechanistically, Filamin assists in constructing the cellular cytoskeleton, where its concentration influences cell adhesion and the migratory capacity of immune cells. The multimeric E3 ubiquitin ligase complex recognizes filamin A as a target protein, and Asb2 encodes the specificity component of this complex, facilitating its recognition of filamin A. AHR upregulates the expression levels of Asb2, consequently modulating the ubiquitination levels of filamin A, thus affecting the migratory capacity of NK cells [84].

NK cell penetration into tumor tissue is inhibited by ubiquitination, which creates an immunosuppressive microenvironment. One important element of the SAGA/STAGA complex is the deubiquitinase USP22. Through ATXN7L3, another component of the nuclear SAGA/STAGA complex, USP22 regulates the tumor immune microenvironment. The levels of NK cells are increased when ATXN7L3 is knocked down [85].

Small molecules regulate the ubiquitin-proteasome pathway in NK cells. A new proteasome inhibitor called b-AP15 prevents the 19 S regulatory particle of the proteasome from deubiquitinating substrates, which inhibits proteasomal substrate breakdown. But unlike bortezomib, the 20 S core proteasome’s proteolytic efficiency is not hindered, which may prevent interference with the capacity of the immunoproteasome to digest antigenic peptides and deliver antigens. When treated with b-AP15, TRAIL-R2 is upregulated, increasing NK cell cytotoxicity [86]. Chlorambucil promotes the degradation of PD-L1 in tumors via activating the GSK3β/β-TRCP signaling pathway. Additionally, chlorambucil significantly increases the amount of NK cells, synergizing with PD-L1 antibodies through interaction with NK cells [87].

**Fig. 8:**
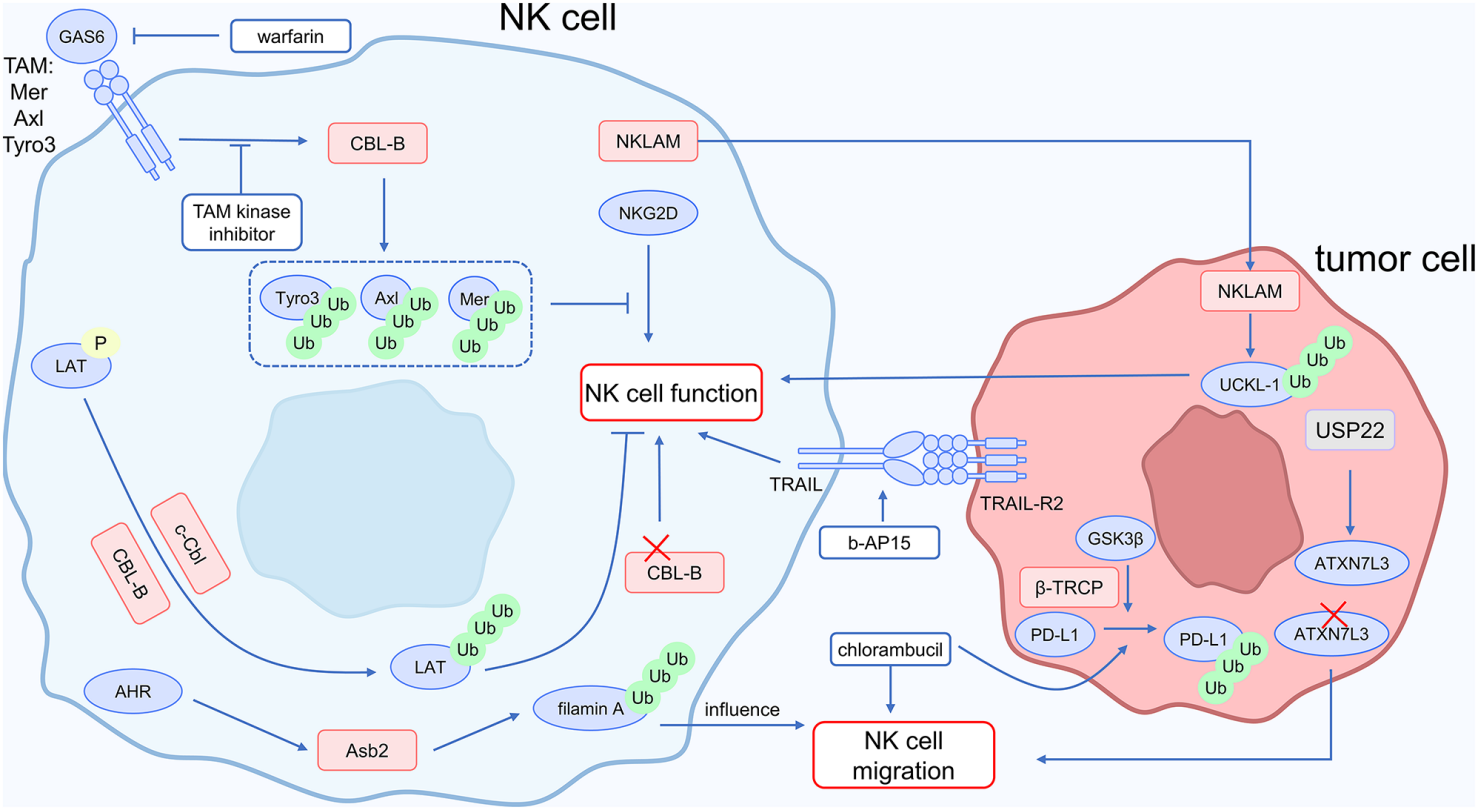
Ubiquitin and NK cells in cancer immunotherapy

Figure 8 illustrates the interaction between NK cells and tumor cells, as well as the mechanism by which ubiquitin modification affects NK cell function. To summarize, E3 ubiquitin ligases such as CBL-B regulate NK cell migration and function. Targeting the ubiquitination pathway in NK cells may help overcome resistance to cancer immunotherapy.

## The interaction between tumor cells and immune cells, as well as the tumor microenvironment, mediates resistance to immunotherapy

### Interaction between tumor and immune cells affects immunotherapy: abnormal MHC antigen presentation

The presentation of the MHC-I antigen is essential to CD8^+^ T cell activity. The poor immunogenicity of tumors and resistance to immunotherapy are linked to MHC-I abnormalities [88]. Ubiquitination modification regulates the protein levels of MHC-I at multiple stages, influencing the presentation of tumor antigens. Targeting the ubiquitination modification process may enhance the effectiveness of tumor immunotherapy. The subsequent examples highlight how ubiquitination modification regulates MHC-I levels.

T-cell immune evasion induced by abnormal MHC-I antigen presentation is one reason for immunotherapy resistance. In acute myeloid leukemia (AML), the sushi domain containing 6 (SUSD6) interacts with transmembrane protein 127 (TMEM127) and MHC-I, recruiting the E3 ubiquitin ligase WWP2. This interaction facilitates WWP2 interaction with MHC-I, leading to its ubiquitination. Targeting TMEM127 can restore MHC-I expression in tumors and inhibit tumor development [89]. Prolonged interferon stimulation leads the PTIR1 gene to be upregulated, which inhibits the production of MHC-I in tumor cells. Mechanistically, one of the ISGs, DDX58 (RIG-I), undergoes alternative splicing in response to prolonged IFN-γ stimulation, in the presence of ADAR-p110, generating the PTIR1 isoform of DDX58. PTIR1 colocalizes with proteasome subunits such as PSMB4 in the cytoplasm, impairing proteasome function. Furthermore, PTIR1 interacts with the deubiquitinase UCHL5, increasing its deubiquitination capacity. This suppresses immunoproteasome activity, which impairs the presentation of new antigens and affects T cell-mediated antitumor immunity [90]. The stability of MHC-I complexes is enhanced by the antigen peptide-loading complex (PLC). TRIM27 induces K48-linked ubiquitination of PLC components, as well as tumor suppressors Rb and p53, promoting their degradation and compromising immune surveillance. The expression levels of the lncRNA LINK-A and the MHC-I protein are negatively correlated. Mechanistically, LINK-A binds to PtdIns(3,4,5)P3, facilitating the interaction between PtdIns(3,4,5)P3-GPCRs and Gαi-GPCRs, reducing intracellular cAMP levels. This affects PKA activity, reducing PKA-mediated phosphorylation of the E3 ligase TRIM27 and enhancing the ubiquitination of the PLC [91]. LINC00624, a long non-coding RNA, inhibits the presentation of MHC I, reducing the infiltration of CD8^+^ T cells in tumors and mediating immune suppression. Adenosine deaminase RNA specific 1 (ADAR1) can edit LINC00624, modifying adenosines in its ADAR1 Editing Region (AER) to inosines, which results in immune suppression. The edited form of LINC00624 suppresses β-TrCP-mediated ubiquitination and degradation of ADAR1, thereby promoting its stability. Furthermore, the edited LINC00624 can suppress the transcription of antigen presentation-related genes in vivo, thereby further promoting immune suppression [92].

The principles of ubiquitination modification can also be harnessed to artificially enhance antigen presentation. Proteolysis-targeting chimeras (PROTACs) that mediate acute drug-induced protein degradation can increase the MHC-I presentation of the degraded proteins. For instance, after the degradation of GFP-S8L-F12 mediated by the small molecule dTAG-7, there is a significant enhancement in the MHC-I antigen presentation of the derived product S8L [93].

Although MHC-II is typically considered to be expressed on antigen-presenting cells as part of the antigen presentation complex, multiple tumor tissues also express MHC-II. Increased expression of tumor-specific MHC-II (tsMHC-II) improves The ability of the immune system to identify tumors. Upregulation of tsMHC-II may represent an innovative method for improving anti-tumor immunity [94]. Another study suggests that MHC-II level in tumor tissues is positively correlated with the effectiveness of immunotherapy [95]. Therefore, ubiquitination modification contributes to the efficacy of immunotherapy by regulating MHC-II expression levels.

The expression of MHC-II in tumors is negatively correlated with the E3 ubiquitin ligase FBXO11. In a dose-dependent manner, FBXO11 blocks the activation of the MHC-II promoter triggered by IFN-γ. Mechanistically, CIITA (MHC class II transactivator) stimulates MHC-II transcription. Through its P/S/T domain, FBXO11 binds to CIITA, enabling the degradation of CIITA [96]. The E3 ubiquitin ligase MDM2 binds to the tumor suppressor protein P53, mediating its degradation. Inhibitors of MDM2, such as Nutlin-3, exhibit anti-tumor effects. Additionally, Nutlin-3 can act as an immune adjuvant by inducing the expression of HLA-I and HLA-DR in tumors through the CIITA, thereby augmenting the anti-tumor properties of T cells [97].

In summary, focusing on the MHC-I ubiquitination and degradation pathway is one possible tactic to improve the effectiveness of immunotherapy. Additionally, promoting MHC-II expression mediated by CIITA in tumor cells may serve as a novel immunotherapeutic approach, although further research is needed to validate this possibility.

### The interaction between tumor and immune cells affects immunotherapy: the inhibitory immune checkpoint PD-L1

Poor tumor response to PD-L1 immunotherapy occurs when there is an absence of PD-L1 [98]. Nevertheless, elevated PD-L1 expression facilitates immune suppression. Thus, additional research is required to elucidate the precise influence of PD-L1 levels on immunotherapy outcomes. Considering the involvement of ubiquitination modification at different stages of PD-L1 expression, targeting the ubiquitin pathway emerges as a promising strategy to enhance the effectiveness of cancer immunotherapy (Table 1).

In the ubiquitin-proteasome degradation pathway, E3 ubiquitin ligases play a direct role in regulating the stability of PD-L1 protein levels. E3 ubiquitin ligase STUB1 facilitates the ubiquitination and subsequent degradation of PD-L1. CMTM6 inhibits this process to stabilize PD-L1 [99]. The E3 ubiquitin ligase SPOP regulates the stability of PD-L1. SPOP binds to PD-L1, leading to the degradation of PD-L1. B cell lymphoma-2-associated transcription factor 1 (BCLAF1) interacts with SPOP, inhibiting the interaction between SPOP and PD-L1, thereby stabilizing PD-L1. The efficacy of immunotherapy is more pronounced in tumor cells with high expression of BCLAF1 [100]. In colorectal cancer (CRC) cells, ALDH2 competes with SPOP for binding to PD-L1, thereby inhibiting the degradation of PD-L1. Inhibiting ALDH2 promotes the response of CRC cells to PD-1 antibody therapy. Since aldehyde dehydrogenase 2 (ALDH2) is involved in alcohol metabolism, alcohol cessation may be beneficial for cancer immunotherapy [101]. Retinoic acid-inducible gene-I (RIG-I) competes with SPOP for PD-L1 binding in colorectal cancer, inhibiting the ubiquitination of PD-L1 and contributing to tumor immune evasion [102]. The sodium-glucose cotransporter-2 (SGLT2) co-localizes with PD-L1 in cells. The SGLT2 inhibitor Canagliflozin disrupts the binding between PD-L1 and SGLT2, inducing PD-L1 degradation with the involvement of the E3 ubiquitin ligase Cullin3^SPOP^, thereby inhibiting tumor immune evasion [103].

**Table 1:** Ubiquitin and PD-L1

Names	Category	biological mechanism	Effect on PD-L1	Refs
STUB1	E3	interacts with PD-L1	↓	[99]
CBL-B	E3	T cells deficient in CBL-B resist PD-L1 immunosuppression.	/	[59]
SPOP	E3	interacts with PD-L1	↓	[100] [101] [102] [103] [141]
SPOP	E3	SPOP induces degradation of IRF1, suppressing IRF1-mediated PD-L1 transcription.	↓	[137]
A20	ubiquitin editing enzyme	interacts with PD-L1	↓	[104]
A20	ubiquitin-editing enzyme	mediate ubiquitination and degradation of PHB, promote STAT3 activation and activate PD-L1 transcription.	↑	[135]
CUL4A	E3	interacts with PD-L1	↓	[106]
β-TrCP	E3	interacts with PD-L1	↓	[107]
RBX1	E3	interacts with PD-L1	↓	[108]
UBQLN4	UBL-UBA protein	interacts with PD-L1	↑	[109]
UBR	E3	Induce ubiquitination and degradation of SHP2 in T cells. Inhibition of SHP2 degradation disrupts PD-1/PD-L1 signaling.	/	[110]
TRIM21	E3	interacts with PD-L1	↓	[111] [143]
TRIM29	E3	inhibits the expression of PD-L1 protein by degrading IGF2BP1.	↓	[105]
TRAF6	E3	promote K63-linked ubiquitination of PD-L1 and stabilize its protein level.	↑	[112]
MIB2	E3	mediate K63-linked ubiquitination of PD-L1 at K136 site, promote PD-L1 localization in cell membrane, and lead to immunosuppression.	↑	[113]
OTUB1	DUB	interacts with PD-L1	↑	[114] [115]
OTUB2	DUB	interacts with PD-L1	↑	[116]
CSN5	DUB	interacts with PD-L1	↑	[117]
USP7	DUB	interacts with PD-L1	↑	[118] [119]
USP2	DUB	interacts with PD-L1	↑	[120]
USP8	DUB	interacts with PD-L1	↑	[121]
USP22	DUB	interacts with PD-L1	↑	[122] [123] [124]
NEDD4	E3	interacts with PD-L1	↓	[125]
ARIH1	E3	interacts with PD-L1	↓	[126]
MDM2	E3	interacts with PD-L1	↓	[127]
HUWE1	E3	interacts with PD-L1	↓	[129]
RNF146	E3	RNF146 induces ubiquitination and degradation of PARP1, promotes STAT3 phosphorylation, and enhances PD-L1 transcription.	↑	[136]
CUL3	E3	facilitates ubiquitination and degradation of PHD2, elevating HIF-1α levels and indirectly promoting PD-L1 transcription.	↑	[139]

Casp8 is involved in cell apoptosis, but it is also correlated with PD-L1 levels. In melanoma cells, upregulation of Casp8 leads to increased levels of the ubiquitin editing enzyme A20, promoting PD-L1 degradation. Conversely, in tumor cells with low expression of Casp8, PD-L1 is highly expressed, resulting in significant tumor progression. However, PD-1 antibody therapy is more effective in tumors with low Casp8 expression [104].

Ubiquitination modification indirectly contributes to stabilizing PD-L1 mRNA. TRIM29 promotes immune cell infiltration in the gastric cancer (GC) microenvironment, enhancing anti-tumor immunity. Mechanistically, IGF2BP1 mediates mRNA stability of PD-L1 through m^6^A modification. In GC, TRIM29 interacts with IGF2BP1, inducing K48-linked ubiquitination of IGF2BP1 at Lys440 and Lys450 sites, resulting in the degradation of IGF2BP1 [105].

Certain compounds regulate PD-L1 by affecting ubiquitination modification. PIK-93 is a small molecule that enhances the efficacy of immunotherapy. Mechanistically, PIK-93 strengthens the physical interaction between the E3 ubiquitin ligase Cullin-4 A (CUL4A) and PD-L1, leading to the degradation of PD-L1 [106]. Sulforaphane (SFN), an isothiocyanate prevalent in cruciferous vegetables, exhibits the capacity to decrease PD-L1 levels. Mechanistically, SFN upregulates the expression of the protein β-TrCP, encoded by the BTRC gene. β-TrCP functions as a ubiquitin E3 ligase adaptor protein, leading to the degradation of PD-L1 [107]. 2,5-dimethyl-celecoxib (DMC) inhibits the proliferation of hepatitis B virus (HBV)-related hepatocellular carcinoma (HCC) cells. Mechanistically, HBV induces high expression of immune checkpoint molecules PD-L1 and CD163 in liver cancer cells. DMC activates the AMPK signaling pathway, where phosphorylated AMPK directly binds to PD-L1. With the involvement of the E3 ubiquitin ligase RBX1, this interaction promotes the degradation of PD-L1 [108]. UBQLN4 belongs to the UBL-UBA protein family and participates in protein degradation. Interestingly, it can also stabilize the levels of specific substrates. In melanoma cells, UBQLN4 interacts with PD-L1, stabilizing PD-L1. Albendazole (ABZ) reduces the expression levels of UBQLN4, resulting in the degradation of PD-L1 and promoting anti-tumor immunity [109]. Reportedly, a novel PROTAC links the E3 ubiquitin ligase UBR to SHP2 in T cells, promoting SHP2 degradation. This disrupts the CD47/SIRPα and PD-1/PD-L1 immune inhibitory signals, reshaping the immune microenvironment [110].

Non-coding RNA also regulates the ubiquitination of PD-L1. LINC02418 interacts with the E3 ubiquitin ligase Trim21 and PD-L1. Overexpression of LINC02418 enhances Trim21-mediated ubiquitination of PD-L1, leading to the disruption of PD-L1 stability. In a non-small cell lung cancer (NSCLC) mouse model, the homologous RNA of hsa-LINC02418, Mmu-4930573I07Rik, enhances the efficacy of PD-1 antibodies [111].

K63 ubiquitination and K48 ubiquitination have different effects on the PD-L1. The E3 ubiquitin ligase TRAF6 interacts with PD-L1, promoting K63-linked ubiquitination of PD-L1, which increases PD-L1 levels. USP8 binds to PD-L1, enhancing K48-linked ubiquitination and reducing K63-linked ubiquitination levels, thereby balancing the action of TRAF6 and maintaining PD-L1 stability [112]. Mind bomb homolog 2 (MIB2) regulates the transportation of PD-L1 to the cell membrane in cancer cells, facilitating PD-1/PD-L1 binding. Mechanistically, MIB2 binds to PD-L1 through its ankyrin repeat domain, promoting K63-linked ubiquitination at the K136 site of PD-L1. This ubiquitination enhances the binding of PD-L1 to Ras-related protein 8 (RAB8), facilitating the translocation of PD-L1 to the plasma membrane, thereby mediating immune suppression [113].

Deubiquitinases promote the stability of PD-L1. OTUB1 removes K48-linked ubiquitin chains from PD-L1, inhibiting PD-L1 degradation via the endoplasmic reticulum-associated degradation (ERAD) pathway. Therefore, targeting OTUB1 may be beneficial for immunotherapy [114]. circIGF2BP3 upregulates plakophilin 3 (PKP3) in NSCLC cells. PKP3 binds to the RNA-binding protein FXR1, thereby stabilizing the mRNA level of the deubiquitinase OTUB1. Subsequently, OTUB1 inhibits the degradation of PD-L1 [115]. Otubain-2 (OTUB2) binds to PD-L1 and inhibits its degradation. The compound OTUB2-IN1-1 inhibits the deubiquitinase activity of OTUB2 without affecting the physical interaction between OTUB2 and PD-L1. OTUB2-IN1-1 promotes PD-L1 ubiquitination in a dose-dependent manner and inhibits tumor growth [116]. CSN5 possesses deubiquitination and stabilization functions for PD-L1. Berberine (BBR), a bioactive alkaloid, interacts with the Glu76 residue of CSN5, leading to the inactivation of CSN5. This promotes PD-L1 degradation and T-cell activation [117]. The A11 peptide derived from ANXA1 competitively binds to PD-L1, inhibiting USP7-mediated deubiquitination of PD-L1 and promoting its degradation in various tumor cells. A11 enhances the efficacy of PD-1 antibodies [118]. Inhibiting USP7 activity enhances T cell killing of gastric cancer cells. Mechanistically, in gastric cancer cells, USP7 interacts with PD-L1 to remove its ubiquitin chains, stabilizing PD-L1 [119]. USP2 regulates the K48-linked ubiquitination of the PD-L1 protein at the Lys270 site through its Thr288, Arg292, and Asp293 residues, thereby decreasing its ubiquitination levels. The attenuation of USP2 expression results in the degradation of PD-L1 [120]. In pancreatic cancer, the deubiquitinase USP8 inhibits the degradation of PD-L1. Targeting USP8 may enhance the effectiveness of immunotherapy for pancreatic cancer [121]. USP22 also maintains the stability of PD-L1 through its deubiquitinase activity [122]. CSN5 is another deubiquitinase for PD-L1. It also interacts with USP22 to maintain its stability. Thus, USP22 and CSN5 collaboratively participate in stabilizing PD-L1 [123]. Although the enhancer of EZH2 is a pro-oncogenic molecule, the efficacy of EZH2 inhibitors is limited, partially due to the immunosuppressive effects induced by EZH2 inhibitors. Mechanistically, EZH2 inhibits the transcription of USP22 through its SET domain-mediated histone methylation activity. Inhibiting EZH2 upregulates the expression of USP22, which stabilizes the protein level of PD-L1 by removing its polyubiquitin chains. The combination of EZH2 inhibitors with immune checkpoint inhibitors demonstrates synergistic effects [124].

The stability of PD-L1 is jointly regulated by various post-translational modifications such as phosphorylation, glycosylation, and ubiquitination. In bladder cancer cells, NEDD4 functions as the E3 ubiquitin ligase for PD-L1. The interaction between NEDD4 and FGFR3 results in the phosphorylation of NEDD4, consequently enhancing its ubiquitin ligase activity and promoting the ubiquitination of PD-L1 [125]. ES-072 is an inhibitor of the EGFR that promotes the degradation of PD-L1 in cancer cells. Mechanistically, ES-072 inhibits EGFR activity, resulting in decreased AKT activity and subsequent activation of GSK3α. Unlike GSK3β, which phosphorylates PD-L1 at the extracellular T180A and S184A sites and mediates its degradation through the E3 ubiquitin ligase β-TrCP, GSK3α-mediated phosphorylation of PD-L1 occurs intracellularly. Activated GSK3α drives the phosphorylation of PD-L1 at the Ser279/283 sites in its cytosolic region. This phosphorylation event recruits the E3 ubiquitin ligase Ariadne-1 homolog (ARIH1), leading to the degradation of PD-L1, and enhancing anti-tumor immune responses [126]. Glycosylation modification affects the ubiquitination of PD-L1. NGLY1, a deglycosylating enzyme, inhibits the N-linked glycosylation of PD-L1, while MDM2 mediates the ubiquitination and degradation of non-glycosylated PD-L1. Interestingly, MDM2 facilitates the connection between PD-L1 and NGLY1, further promoting the degradation of PD-L1 [127]. RBMS1, categorized within RNA-binding proteins (RBPs), functions in the regulation of RNA stability. The loss of RBMS1 downregulates the protein levels of PD-L1. Mechanistically, Glycosyltransferase beta-1,4-galactosyltransferase 1 (B4GALT1) is a novel glycosyltransferase that regulates the glycosylation of PD-L1. RBMS1 interacts with the 3′-UTR of B4GALT1 mRNA, stabilizing B4GALT1 mRNA and resulting in an increased expression of B4GALT1. This, in turn, promotes the glycosylation of PD-L1 protein and enhances the stability of PD-L1 protein [128]. HECT, UBA, and WWE domain-containing protein 1 (HUWE1) is an E3 ubiquitin ligase of the PD-L1. Transmembrane and ubiquitin-like domain-containing protein 1 (TMUB1) shares a similar binding ability with PD-L1 as HUWE1. They competitively bind to PD-L1, reducing the degradation of PD-L1. Additionally, TMUB1 recruits STT3A to facilitate the glycosylation modification of PD-L1. This inhibition of ER-associated protein degradation promotes immune evasion in tumors [129].

Other post-translational modifications also participate in regulating the ubiquitination of PD-L1. Interferon-stimulated gene 15 (ISG15) serves as a mediator of a protein translation modification called ISGylation, which is similar to ubiquitination. In lung adenocarcinoma (LUAD), ISG15 engages in K48-modified ISGylation with glycosylated PD-L1, promoting an increase in the ubiquitination levels of PD-L1, causing it to degrade. This enhancement contributes to an improved efficacy of anti-PD-L1 immunotherapy [130]. UFM1 modification (UFMylation) is a distinctive form of protein modification that is quite similar to ubiquitination. Key components required for UFMylation include E1 (UBA5), E2 (UFC1), and E3 (UFL1), along with Ubiquitin-fold modifier 1 (UFM1). In tumor cells, activated UFMylation promotes the ubiquitination of PD-L1, while it has no impact on the glycosylation of PD-L1. Silencing either UFL1 or UFM1 stabilizes PD-L1, disrupting anti-tumor immune responses [131]. In TNBC cells, PLAC8 binds to PD-L1, enhancing the stability of the PD-L1 protein. Knocking down PLAC8 increases the ubiquitination of PD-L1 and reduces its glycosylation. Ubiquitin-fold modifier 1 (UFM1) mediates UFMylation on PLAC8, contributing to the increased stability of the PLAC8 protein [132]. In tumor cells, docosahexaenoic acid (DHA) inhibits the expression of PD-L1. Mechanistically, palmitoylation of PD-L1 inhibits its ubiquitination, maintaining its stability. DHA inhibits palmitoyltransferase DHHC5 in tumors, reducing the palmitoylation level of PD-L1, thereby promoting its ubiquitination. DHA also inhibits the deubiquitinating enzyme CSN5, further enhancing PD-L1 ubiquitination, and reducing its stability [133].

Supplementing copper in cancer cells stabilizes PD-L1 mRNA. Copper chelators downregulate STAT3, inhibiting PD-L1 transcription. Additionally, they also inhibit EGFR signaling, promoting PD-L1 ubiquitination and degradation [134]. In cells, Prohibitin (PHB) inhibits STAT3 phosphorylation. A20 interacts with PHB, ubiquitinates PHB, and induces PHB degradation, thereby promoting STAT3 phosphorylation and activation, enhancing PD-L1 transcription. Knockdown of A20 inhibits STAT3-induced PD-L1 expression, promoting the effectiveness of PD-1 antibodies [135]. Toll-like receptor 9 (TLR9) agonist ODN1585 enhances the efficacy of PD-1 antibody. Mechanistically, STAT3 enhances PD-L1 expression. PARP1 synthesizes poly ADP-ribose (PAR) and binds to STAT3, causing PARylation of STAT3, which significantly inhibits STAT3 phosphorylation. Stimulation of TLR9 leads to autoPARylation of PARP1 mediated by poly ADP-ribose glycohydrolase (PARG), mediating the degradation of PARP1 via RNF146, an E3 ubiquitin ligase. Downregulation of PARP1 levels promotes STAT3 phosphorylation, increasing PD-L1 transcription [136]. As a transcription factor, IRF1 increases PD-L1 expression. In endometrial cancer (EC), the E3 ubiquitin ligase SPOP interacts with IRF1, inducing its degradation, thereby inhibiting PD-L1 transcription. Gene mutations in SPOP increase PD-L1 expression in EC cells [137]. Simvastatin has unexpected effects on regulating tumor immunity. Mechanistically, the long noncoding RNA SNHG29 interacts with the transcription factor Yes-associated protein (YAP), inhibiting YAP phosphorylation to promote its nuclear localization. This, in turn, inhibits YAP degradation through the ubiquitin-proteasome pathway and enhances YAP transcriptional activity. Simvastatin inhibits lncRNA SNHG29, disrupting YAP-mediated PD-L1 transcription [138]. Elevated expression of Bcl-2-associated transcription factor-1 (BCLAF1) restricts the therapeutic efficacy of atezolizumab and bevacizumab. Mechanistically, the interaction between BCLAF1 and CUL3 leads to the degradation of hydroxylase domain protein 2 (PHD2), promoting HIF-1α expression, and indirectly enhancing PD-L1 transcription [139]. In TNBC patients, Transglutaminase 2 (TG2) is associated with resistance to PD-L1 therapy. Mechanistically, TG2 induces transamidation, leading to the crosslinking of tumor suppressor factors such as PTEN and IκBα. This promotes the ubiquitination and degradation of PTEN and IκBα, consequently enhancing PD-L1 expression [140].

The level of PD-L1 is influenced by the cell cycle, increasing from the M phase and starting to decrease from the late G1 phase. A negative correlation exists between the abundance of PD-L1 and the activity of Cyclin-dependent kinase 4 (CDK4). Mechanistically, the E3 ligase adaptor Cdh1 ubiquitinates SPOP, inducing its degradation. Cyclin D-CDK4 kinase directly phosphorylates SPOP at the Ser6 site in Cullin3^SPOP^, leading to the physical binding of SPOP with 14-3-3γ. This interaction reduces the association between SPOP and Cdh1, promoting the stability of SPOP, thereby downregulating PD-L1. CDK4/6 inhibitors enhance the efficacy of PD-L1 antibodies [141]. The transcription factor Myeloid zinc finger 1 (MZF1) promotes the transcription of CDK4. CDK4 also directly binds to MZF1, enhancing the expression levels of MZF1. This positive feedback loop promotes PD-L1 ubiquitination. Inhibiting the levels of MZF1 using CDK4 inhibitors is beneficial for enhancing the efficacy of PD-L1 antibodies [142]. The E3 ubiquitin ligase TRIM21 induces PD-L1 degradation. In LUAD cells, CDK5 interacts with TRIM21. Inhibiting CDK5 expression promotes TRIM21-mediated PD-L1 degradation, enhancing anti-tumor immunity [143].

### Interaction between tumor and immune cells affects immunotherapy: other immune checkpoints

Acquisition of inhibitory immune checkpoints leads to resistance in cancer immunotherapy [2]. Hence, besides PD-L1, targeting other immune checkpoints represents a potential approach to overcoming resistance in cancer immunotherapy. Recent studies indicate that ubiquitination modification regulates various immune checkpoint signaling transduction (Table 2).

CD137 (4-1BB) is a T cell co-stimulatory molecule, that enhancing anti-tumor immune responses. K63-linked ubiquitination enhances protein-protein interactions, facilitating downstream signaling. The CD137-TRAF2 complex activates CD137 downstream signaling in a K63-linked dependent manner. Deubiquitinases A20 and Cylindromatosis (CYLD) interact with the CD137-TRAF2 complex, reducing its ubiquitination level and thus inhibiting CD137 signaling transduction [144].

**Table 2:** Ubiquitin and other immune checkpoint

Names	Category	Immune checkpoint	Effect on immune checkpoint	biological mechanism	Refs
TRIM21	E3	IDO1	↓	ubiquitination of IDO1 and promotion of its degradation.	[158]
USP14	DUB	IDO1	↑	removal of K48-linked ubiquitination from IDO1 to maintain its protein stability.	[158]
TRAF6	E3	CTLA-4	↓	mediate K63-linked ubiquitination of CTLA-4 and promote its degradation.	[160]
TRIP12	E3	PD-1	↓	promote the ubiquitination and degradation of NFATc1 and inhibit the expression of PD-1.	[157]
USP22	DUB	CD73	↑	inhibit that ubiquitination and degradation of CD73.	[151]
CRL4^WDR4^	E3	CD73	↑	induced ubiquitination and degradation of PML, and induced expression of CD73.	[150]
TRIM21	E3	CD73	↓	induce ubiquitination and degradation of CD73.	[149]
AMFR	E3	B7-H4	↓	induce ubiquitination and degradation of B7-H4.	[148]
TRIM21	E3	CD47	↓	induced ubiquitination and degradation of CD47 at K99/K102 sites.	[146]
A20	DUB	CD137-TRAF2 complex	↓	inhibit the level of K63-linked ubiquitination of CD137-TRAF2 complex and its function.	[144]
CYLD	DUB	CD137-TRAF2 complex	↓	inhibit the level of K63-linked ubiquitination of CD137-TRAF2 complex and its function.	[144]
FBXO38	E3	PD-1	↓	mediate ubiquitination and degradation of PD-1 at Lys233 site.	[154]
USP5	DUB	PD-1	↑	deubiquitination and stabilization of PD-1.	[156]
FBW7	E3	PD-1	↓	mediates K48-linked ubiquitination and degradation of the K233 residue of PD-1.	[152]
KLHL22	E3	PD-1	↓	KLHL22, CUL3, and RBX1 form a complex, promoting K210 and K233 site ubiquitination of PD-1 and facilitating the degradation of PD-1.	[153]

The immune checkpoint CD47 is highly expressed in tumors, inhibiting tumor antigen presentation and mediating immune suppression. The aqueous extract of Taxus chinensis var. mairei (AETC) promotes ubiquitination and degradation of CD47 in tumor cells, activating immune cells and enhancing the efficacy of PD-1 antibodies [145]. In tumor cells with high expression of EGFR, the expression levels of CD47 are also upregulated, leading to enhanced growth and immune evasion capabilities. Mechanistically, EGFR activates c-Src, which phosphorylates CD47 at the Y288 site. TRIM21 induces ubiquitination and degradation of CD47 at the K99/K102 sites. Phosphorylation of CD47 inhibits TRIM21-mediated ubiquitination, stabilizing CD47 protein levels. Combination therapy of anti-CD47 antibodies with EGFR inhibitors demonstrates more effective inhibition of tumor growth [146].

The co-stimulatory receptor CD28 participates in T cell activation. CD8^+^ T cells with CD28 defects often accumulate in tumors, leading to adverse clinical outcomes. CD28 deficiency also impairs the efficacy of PD-1 antibodies. Lenalidomide stimulates CD28-deficient T cells, emulating the transcriptional changes induced by CD28 stimulation and activating T cells. Mechanistically, IL-2 and Notch are downstream factors in T cell CD28 co-stimulatory signal transduction, IKZF1 and IKZF3 inhibit IL-2 and Notch signaling. In CD28-deficient cells, lenalidomide, mediated by the E3 ubiquitin ligase CRL4^CRBN^, induces the degradation of IKZF1 and IKZF3, leading to increased IL-2 expression, enhanced Notch signaling transduction, and improved efficacy of PD-1 antibodies [147].

The level of B7-H4 is associated with tumor immune evasion. Autocrine motility factor receptor (AMFR), an E3 ubiquitin ligase, ubiquitinates B7-H4. STT3A and UGGG1 are specific glycosyltransferases that catalyze the glycosylation of B7-H4, antagonizing its ubiquitination degradation. Targeting the immune inhibitory molecule B7-H4 enhances the immunogenicity of cancer cells [148].

CD73 generates adenosine, inhibiting the function of T cells. TRIM21 ubiquitinates CD73 and promotes its degradation. The overexpression of TRIM21 in tumor cells reduces adenosine production, resulting in increased expression of IFN-γ and promoting T-cell proliferation. Stimulation by IFN-γ further elevates the protein levels of TRIM21, creating a positive feedback loop termed the IFNγ-TRIM21-CD73 loop, which further decreases the levels of CD73 [149]. The E3 ubiquitin ligase WD repeat 4–containing cullin-RING ubiquitin ligase 4 (CRL4^WDR4^) induces ubiquitination and degradation of Promyelocytic leukemia (PML) and promotes the expression of CD73. This leads to an increase in Tregs and M2-like macrophages and a decrease in CD8^+^ T cells. Blocking CD73 can reverse this immunosuppressive effect [150]. In breast cancer, the deubiquitinating enzyme USP22 interacts with CD73, inhibiting CD73 ubiquitination and proteasomal degradation, thereby stabilizing its protein levels. Inhibiting USP22 reduces breast cancer progression [151].

PD-1, an inhibitory immune checkpoint, is regulated by ubiquitination modification. F-box and WD repeat domain containing 7 (FBW7) is an E3 ubiquitin ligase that directly interacts with PD-1 in the nucleus, facilitating its degradation. Cyclin-dependent kinase 1 (CDK1) phosphorylates PD-1, promoting its nuclear localization and subsequent ubiquitination. The effectiveness of PD-1 antibodies is influenced by FBW7 upregulation [152]. The complex formation involving E3 ligase KLHL22, CUL3, and RBX1 ubiquitinates PD-1 with incomplete glycosylation at the K210 and K233 sites, ultimately promoting the degradation of PD-1 [153]. The E3 ubiquitin ligase FBXO38 mediates the degradation of PD-1. FBXO38 is transcriptionally suppressed in tumor-infiltrating T cells. Supplementing IL-2 rescues FBXO38 transcription, downregulating PD-1 in T cells [154]. Core fucosylation catalyzed by Core Fucosyltransferase (Fut8) is a common glycosylation modification in tumors. Knocking down Fut8 in Jurkat cells results in the loss of core fucosylation, promoting the degradation of PD-1 induced by FBXO38 [155]. Extracellular signal-regulated kinase (ERK) phosphorylates PD-1, promoting its interaction with USP5 and stabilizing PD-1, thereby facilitating immune evasion. Inhibiting ERK or USP5 can suppress this immune evasion and activate CD8^+^ T cells [156].

Ubiquitination modification also regulates the inhibitory immune checkpoint IDO1. Interestingly, the immunosuppressive effects of IDO1 are typically associated with PD-1. IDO inhibitors lead to the accumulation of tryptophan within T cells, resulting in PD-1 expression. Mechanistically, NFATc1 promotes PD-1 expression, and overexpression of tryptophanyl-tRNA synthetase (WARS) increases intracellular protein tryptophanylation levels through lysine tryptophanylation. After lysine residues at position K1136 in the E3 ubiquitin ligase TRIP12 undergo tryptophanylation, enhanced binding between TRIP12 and NFATc1 occurs. This promotes ubiquitination and degradation of NFATc1, leading to reduced PD-1 protein expression. NAD+-dependent sirtuin 1 (SIRT1) can remove tryptophan from TRIP12. Overexpression of SIRT1 increases PD-1 expression [157]. One of the reasons for the poor efficacy of IDO1 inhibitors is the activation of the aryl hydrocarbon receptor (AhR), which exacerbates immune suppression. The E3 ubiquitin ligase TRIM21 ubiquitinates IDO1, while the deubiquitinating enzyme USP14 interacts with IDO1 through its ubiquitin-like domain (UBL), removing its K-48 linked ubiquitination and maintaining protein stability. Targeting USP14 downregulates IDO1 protein levels, enhances sensitivity to PD-1 antibodies, and does not activate AhR signal transduction [158]. In colorectal cancer cells, the expression of HIF1α increases in the hypoxic tumor microenvironment. The hypoxic tumor microenvironment of colorectal cancer induces HIF1α expression, thereby increasing FSTL3 levels. FSTL3 binds to c-Myc and reduces its ubiquitination levels, stabilizing c-Myc. c-Myc induces the expression of PD-L1 and IDO1, promoting tumor immune evasion. Elevated FSTL3 expression induces resistance to anti-PD immunotherapy, while combining PD-1 antibodies with IDO1 inhibitors exhibits enhanced therapeutic efficacy for tumors with high FSTL3 expression [159].

Ubiquitination modification also regulates the immune checkpoint CTLA-4. Within T cells, TRAF6 facilitates the ubiquitination of CTLA-4 at Lys63 sites, consequently fostering CTLA-4 degradation through the lysosome, ultimately enhancing anti-tumor immunity [160].

### Immunosuppressive tumor microenvironment: MDSC

Myeloid-derived suppressor cells (MDSCs) participate in the formation of resistance to immunotherapy [1]. The ubiquitination pathway regulates the recruitment of MDSCs. In pancreatic cancer tissues with low USP22 expression, the number of granulocytic myeloid-derived suppressor cells (gMDSCs) decreases, while the numbers of CD4^+^ T cells and CD8^+^ T cells increase. This leads to an enhancement in the efficacy of immunotherapy [85]. AK036396, a long non-coding RNA enhances the immune regulatory capacity of polymorphonuclear myeloid-derived suppressor cells (PMN-MDSCs). Mechanistically, the immunosuppressive function of PMN-MDSCs relies on Ficolin B (Fcnb), and AK036396 directly interacts with Ficolin B (Fcnb), preventing its ubiquitination degradation and enhancing its stability. Consequently, PMN-MDSC maturation is inhibited, promoting immune suppression. Moreover, inhibition of lncRNA AK036396 decreases the activity of the immunosuppressive molecule Arginase 1 (Arg1) [161]. In regulating the proliferation and activation of MDSCs, the transcription factor STAT3 plays a crucial role. TRAF6 binds to STAT3 in MDSC cells, increasing STAT K63-linked ubiquitination and phosphorylation levels, thereby promoting MDSC-mediated immunosuppression [162]. Autophagy serves as a pivotal pathway in the MDSC-mediated anti-tumor immune suppression. MDSCs with impaired autophagy exhibit compromised lysosomal degradation of MHC II molecules. This defect leads to a more efficient presentation of tumor antigens. Moreover, E3 ubiquitin ligase membrane-associated RING-CH1 (MARCH1) ubiquitinates MHC II molecules in monocytic MDSCs (M-MDSCs), facilitating their degradation through the lysosomal pathway. By inhibiting MARCH1, the levels of MHC II molecules are significantly increased, leading to enhanced anti-tumor immune responses [163].

Ubiquitination modification can regulate the function of MDSCs by modulating the NF-κB signaling pathway. Receptor-interacting protein kinase 1 (RIPK1) undergoes K63-linked ubiquitination mediated by the E3 ligase TRIM28, leading to activation of the NF-κB pathway. Activation of NF-κB leads to the expression of CXCL1, recruiting MDSCs and forming an immunosuppressive microenvironment [164]. WDR6 increases the abundance of MDSCs in the tumor, forming an immunosuppressive microenvironment. Mechanistically, the UV radiation resistance associated gene (UVRAG) is involved in mediating autophagy. Mediated by the WDxR motif, WDR6 interacts with the E3 ubiquitin ligase CUL4A/DDB1 complex and UVRAG, leading to the ubiquitination of UVRAG at the K176 site, promoting UVRAG degradation and disrupting autophagy. The disruption of autophagy by WDR6 prevents the autophagic degradation of one member of the NF-κB family, p65, leading to its interaction with TNFα and promoting TNFα expression. TNFα, in turn, promotes the transcription of WDR6. The WDR6/TNFα signaling promotes TNFα-mediated recruitment of MDSCs. Disrupting the binding between WDR6 and UVRAG using WDxR-like peptides inhibits MDSC-mediated immune suppression and enhances the efficacy of PD-L1 antibodies [165]. The deubiquitinase USP12 maintains p65 protein levels, activating the p65/NF-κB signaling pathway, leading to increased expression of PD-L1 and inducible nitric oxide synthase (iNOS), promoting MDSC-mediated immune suppression [166].

In addition to the aforementioned ways of affecting MDSC recruitment and function, potential strategies to reverse MDSC-mediated immunotherapy resistance also include: inhibiting key molecules involved in MDSC-mediated immunosuppression such as COX2, ARG1, iNOS, and TGF-β; targeting STAT3 to suppress MDSC accumulation; inducing MDSC apoptosis by targeting molecules on the MDSC cell membrane, such as CD33; and inhibiting cytokine signaling pathways that recruit MDSCs [167]. The implementation of these strategies may benefit from targeting ubiquitination modification.

### Immunosuppressive tumor microenvironment: TAM

Tumor-associated macrophages (TAMs) are classified into two types, among which M2 TAMs exhibit higher levels of PD-L1. As the tumor progresses, the number of PD-L1 + M2 TAMs continues to increase. M2 TAMs are involved in mediating immunotherapy resistance [168]. Ubiquitination modifications influence TAM reprogramming and participate in TAM-mediated immunosuppression.

In macrophages with high expression of c-Myc, the expression of M2 markers such as Arg-1 and TGF-β is increased. Aminoacyl tRNA synthase complex-interacting multifunctional protein 2 (JTV-1) binds to FUBP1 protein and mediates its degradation via the ubiquitin-proteasome pathway. Long non-coding RNA NR_109 competes with JTV-1 for binding to FUBP1, inhibiting FUBP1 degradation. FUBP1 enhances c-Myc transcription, and c-Myc recognizes the NR_109 promoter, promoting NR_109 transcription. This positive feedback further promotes the M2 polarization of TAMs [169]. Another study suggests that c-Myc is ubiquitinated by the FBXW7, resulting in suppressed generation of M2 TAMs [170]. USP18 regulates the function of the type I interferon (IFN-I) pathway. In USP18-deficient macrophages, the protein level of Colony Stimulating Factor 1 Receptor (CSF1R) is downregulated, promoting TAM-mediated immunosuppression. Mechanistically, the E3 ubiquitin ligase NEDD4 binds to CSF1R, facilitating its degradation. USP18 interacts with NEDD4, inhibiting NEDD4-mediated ubiquitination of CSF1R, thereby reducing CSF1R degradation. Additionally, in USP18-deficient macrophages, IFN-I induces the E2 ubiquitin-conjugating enzyme UBCH5, resulting in the downregulation of CSF1R levels [171]. FATS (fragile site-associated tumor suppressor) is an E3 ubiquitin ligase, and knocking down FATS can promote macrophage polarization towards the M1 phenotype. Mechanistically, FATS inhibits the phosphorylation of IκBα to suppress its K48-linked ubiquitination, promoting the accumulation of IκBα in the cytoplasm and inhibiting NF-κB transcription. Deletion of FATS leads to activation of the NF-κB signaling pathway, which promotes increased expression of macrophage MHC-II and secretion of pro-inflammatory cytokines. This ultimately results in the polarization of macrophages towards the M1 phenotype, activating T cells to inhibit tumor progression [172]. In mice, inhibition of USP7 activates the p38-MAPK signaling pathway, promoting the transformation of TAMs into the M1 phenotype and inhibiting tumor progression [173].

Tumor-associated macrophages (TAMs) release exosomes containing non-coding RNAs, which affect the ubiquitination of MHC-I in tumor cells, thereby promoting tumor immune evasion. TAMs transfer LINC01232 to tumor cells through exosomes. LINC01232 facilitates the nuclear translocation of E2F2, promoting its binding to the NBR1 promoter and enhancing NBR1 transcription. Under autophagy-lysosome signaling, NBR1 promotes the ubiquitination-mediated degradation of MHC-I, impairing anti-tumor immunity [174]. Another analogous study suggests that LINC01592 secreted by M2-TAMs binds to E2F6 in tumor cells, enhancing NBR1 transcription, leading to MHC-I degradation and defective anti-tumor immunity [175].

**Fig. 9:**
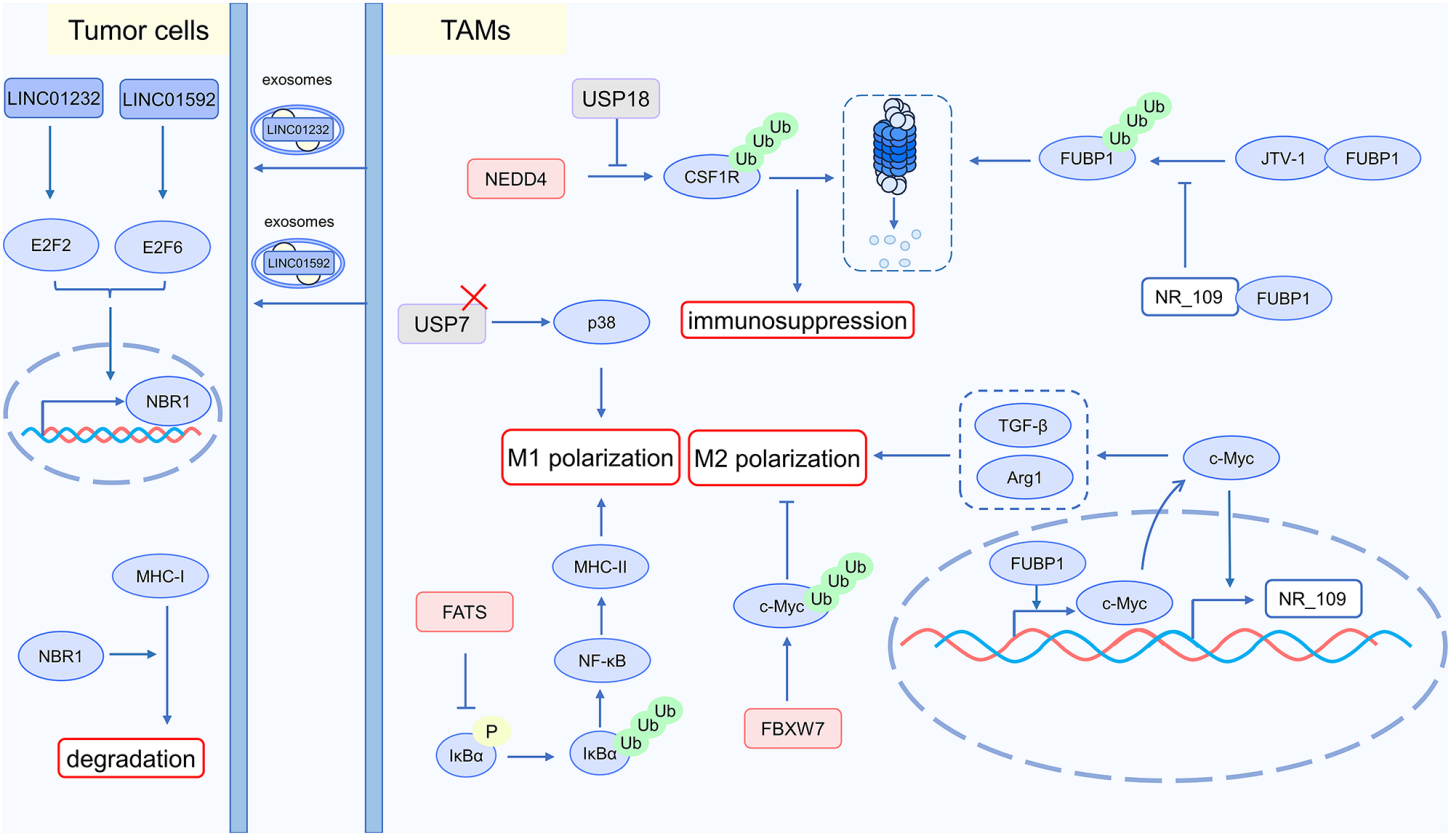
Ubiquitin and TAMs in cancer immunotherapy

The mechanism by which ubiquitin modification regulates the function of TAMs is illustrated in Fig. 9. In summary, targeting ubiquitination to reprogram M2 TAMs into M1 TAMs is a feasible approach to overcome resistance to cancer immunotherapy. Targeting ubiquitination to increase tumor MHC-I expression levels is another approach to overcome immune suppression mediated by M2 TAMs.

### Immunosuppressive tumor microenvironment: Treg

The function of cytotoxic T cells is modulated by regulatory T cells (Treg cells), which also create an immunosuppressive environment and encourage resistance to immunotherapy [1]. Ubiquitin modification regulates the immunosuppressive function of Treg cells by affecting the levels of transcription factors such as Foxp3.

The transcription factor Foxp3 is important for preserving the suppressive function of Tregs. Its expression is finely regulated by both the Usp22 and the E3 ligase Rnf20. Mechanistically, the loss of USP22 increases the ubiquitination level of Foxp3. Additionally, USP22 regulates the transcription of Foxp3. Knockdown of USP22 increases the ubiquitination of histone H2B at the K120 site in Treg cells, activating a series of histone modifications, including H3K4me and H3K27ac, affecting various gene regulatory elements, including Treg-specific super-enhancers. E3 ubiquitin ligase Rnf20 can bind to Foxp3 and cooperatively regulate Foxp3 levels with USP22. Targeting Rnf20 reduces the ubiquitination level at the H2B K120 site, while low expression of USP22 increases H2B ubiquitination levels [176]. TRAF6, an E3 ubiquitin ligase, enhances the K63-linked ubiquitination of Foxp3. The absence of K63 modification on Foxp3 results in abnormal nuclear localization, impairing the proper regulation of Treg gene transcription by Foxp3. This disruption affects the function and stability of Tregs [177]. Heat shock proteins (HSPs), serving as molecular chaperones, enhance immune responses under various stress conditions. HSP70 interacts with Foxp3, recruiting the E3 ubiquitin ligase Stub1 to encourage the degradation of Foxp3 [178]. TGF-β stimulation in Treg cells elevates USP44 levels. USP44, through its ZF-proline-rich domain, interacts with the proline-rich domain of FOXP3, removing its ubiquitin chains and stabilizing the protein levels of FOXP3. Additionally, USP44 collaborates with USP7 to collectively remove ubiquitin chains from FOXP3, further promoting the stability of FOXP3 protein levels [179]. USP7 facilitates the formation of Foxp3 homodimers. Inhibiting USP7 suppresses Treg activity, thereby enhancing anti-tumor immunity [180]. TCR activation, when combined with Ca^2+^, activates TGF-β Activated Kinase 1 (TAK1). TAK1 stimulates the activity of Nemo-Like Kinase (NLK), a member of the MAPK family. NLK induces Foxp3 phosphorylation, increasing the stability of the Foxp3. Mechanistically, when NLK is overexpressed, it prevents Foxp3 from being degraded by E3 ubiquitin ligase STUB1, which raises Foxp3 protein levels [181].

Ubiquitination modification regulates the function of Tregs by influencing transcription factors. The deficiency of the transcription factor Helios (IKZF2) hinders the immunosuppressive activity of Tregs. Transcription factors have traditionally been considered challenging drug targets; however, inducing ubiquitination and proteasomal degradation of transcription factors provides a potential pharmacological approach for targeting them. Through pharmacological reprogramming of the E3 ubiquitin ligase CUL4-DDB1-RBX1-CRBN (CRL4^CRBN^), a compound named ALV2 has been developed. ALV2 binds to CRBN, inducing CRBN-Helios dimerization and selectively degrading Helios. ALV2 attenuates the immunosuppressive capacity of human Treg cells [182]. The Rnf18 gene-encoded E3 ligase Gene Related to Anergy in Lymphocytes (GRAIL) causes the TCR-CD3 complex to get ubiquitinated and degraded in the endosomal compartment, which in turn suppresses the production of NFATc1. NFATc1 regulates the transcription of IL-21, and knocking down Rnf18 increases the expression of both NFATc1 and IL-21. Elevated expression of IL-21 leads to functional defects in Treg cells [183].

Interestingly, CBL-B seems to be an important modulator of Treg activity. Lacking CBL-B, CD8^+^ T cells express an excessive amount of IFN-γ and are resistant to Treg suppression [184]. Functioning as a negative regulator within the TCR signaling pathway, Cbl-b modulates immune responses. CD4^+^ T cells deficient in Cbl-b exhibit elevated IL-2 production, thereby escaping the immunosuppression imposed by Treg cells. IL-2 plays an important role in overcoming cytokine-induced resistance in Treg cells [185].

To summarize, inhibiting CBL-B or modulating Foxp3 levels via ubiquitination represents potential approaches to disrupt the immunosuppressive function of Tregs. Furthermore, blocking certain cytokine signaling pathways, such as CCR4, leads to impairment of the immunosuppressive effects mediated by Tregs [1]. Intervening in ubiquitination modification represents a potential approach to block these cytokine pathways.

## Ubiquitin modification and CAR-T

Ubiquitin modification also influences the therapeutic efficacy of CAR-T cells. The limited in vivo persistence of CAR-T cells hampers therapeutic efficacy, and this is associated with rapid ubiquitination upon CAR binding to tumor antigens, followed by subsequent lysosomal degradation. Significantly inhibiting antigen-stimulated CAR ubiquitination and suppressing CAR downregulation, the substitution of all lysine residues in the cytoplasmic domain of CAR (CARKR) with arginine enhances the long-term anti-tumor capabilities of CAR-T cells [186]. In exhausted CAR-T cells, there is a high expression of the E3 ubiquitin ligase CBL-B. Deleting CBL-B in CAR-T cells leads to increased expression of IFN-γ and TNF-α, thereby enhancing their tumor-killing abilities. Importantly, this genetic modification does not show signs of inducing autoimmune abnormalities [64]. The anti-tumor efficacy of CAR-T cells is enhanced by sulforaphane (SFN). Mechanistically, SFN suppresses the activity of the PI3K/AKT signaling pathway, resulting in reduced levels of PD-L1. SFN also promotes the expression of the BTRC gene, increasing the protein levels of β-TrCP. As an E3 ubiquitin ligase, β-TrCP facilitates the ubiquitination process leading to the degradation of PD-L1, thereby inhibiting the IFN-γ-induced upregulation of PD-L1 expression [107]. Protein degraders developed based on E3 ubiquitin ligases serve to bridge target proteins and E3 ubiquitin ligases, inducing the degradation of target proteins. In CAR-T cells, utilizing such protein degraders to promote the degradation of SMAD2 and SMAD3 proteins can block SMAD-dependent TGF-β signaling, overcoming TGF-β-induced T cell dysfunction and enhancing CAR-T cell antitumor capabilities. Compared to CRISPR/Cas9-based gene knockout, protein degraders offer a more straightforward approach in practical use, minimizing additional steps in the manufacturing process of cell therapy products [187].

## Research progress of drugs targeting ubiquitin signaling: targets, compounds, and clinical trials

Currently, many small molecule inhibitors targeting key components of ubiquitin-related signaling pathways are being developed as novel approaches for cancer treatment. Among them, inhibitors of the E3 ubiquitin ligase MDM2 are a major focus of research.

In orthotopic glioblastoma (GBM) patient-derived brain tumor stem cell (BTSC) xenograft models, treatment with the MDM2 small molecule inhibitor brigimadlin (BI-907828) showed higher survival rates compared to the control group [188]. BI-907,828 is currently undergoing Phase II clinical trials (NCT05512377) to evaluate its potential as a second-line treatment for advanced cholangiocarcinoma, bladder cancer, and other solid tumors [189]. In another clinical trial (NCT03449381), Phase Ia results demonstrated the preliminary efficacy of BI-907,828/brigimadlin in solid tumors, with particularly notable efficacy in liposarcoma [190]. BI-907,828 is being evaluated in a Phase III clinical trial (NCT06058793) to further determine its efficacy in treating dedifferentiated liposarcoma (DDLPS) [191].

Interestingly, another MDM2 inhibitor, APG-115, has a unique role in enhancing anti-tumor immunity, offering hope for patients resistant to immunotherapy. By targeting MDM2, APG-115 not only inhibits tumor growth but also reduces the apoptosis of tumor-infiltrating CD8^+^ T lymphocytes. The anti-tumor effect of APG-115 depends on CD8^+^ T cells; in mouse models, depletion of CD8^+^ T cells suppresses the anti-tumor activity of APG-115 [192]. In syngeneic tumor models, APG-115 administration reduced M2 macrophage infiltration, disrupted the immunosuppressive tumor microenvironment, and reversed tumor resistance to immune checkpoint inhibitors. The combination of APG-115 and PD-1 antibodies demonstrated enhanced anti-tumor activity, which was independent of the p53 status of tumor cells but depended on the activation of p53 in immune cells [193]. Not only in mouse models, but a Phase II clinical trial (NCT03611868) has also further confirmed the ability of APG-115 to reverse resistance to immunotherapy. For patients with refractory cutaneous melanoma whose disease progressed after PD-L1/PD-1 antibody treatment, the combination of APG-115 (alrizomadlin) and pembrolizumab showed efficacy, with an overall response rate (ORR) of 23.1%. This study is ongoing to evaluate the efficacy of APG-115 as a monotherapy [194]. Additionally, in a Phase I clinical trial (ChinaDrugTrials.org.cn: CTR20170975), APG-115 demonstrated anti-tumor activity against advanced solid tumors, with significant efficacy in tumors that are TP53 wild-type and have MDM2 amplification [195].

Navtemadlin (AMG-232) is another MDM2 inhibitor. In mouse models, navtemadlin has shown efficacy against EBV-positive lymphomas, inducing tumor regression and inhibiting the systemic metastasis of tumor cells [196]. Navtemadlin (AMG-232) also has the ability to modulate anti-tumor immunity. In co-culture systems of T cells and tumor cell lines, the combination of navtemadlin and PD-1 antibodies enhanced T cell-mediated tumor killing [197]. Additionally, two other MDM2 inhibitors are currently undergoing Phase I clinical trials: ASTX295 (NCT03975387) and siremadlin (NVP-HDM201) (NCT05155709). Both have shown potential as anti-tumor agents [198, 199].

Despite many encouraging results, the clinical application of MDM2 inhibitors in cancer treatment still faces numerous challenges. Unfortunately, in a completed Phase III clinical trial (NCT02545283), the combination of the MDM2 inhibitor idasanutlin (RG-7388) and cytarabine did not improve overall survival in patients with relapsed or refractory acute myeloid leukemia (AML) [200]. Additionally, MDM2 inhibitors may have adverse effects, such as promoting p53 mutations and generating various MDM2 protein isoforms. Severe adverse reactions, including thrombocytopenia or neutropenia, are also concerns when using MDM2 inhibitors [201].

In addition to MDM2, another E3 ubiquitin ligase, CBL-B, has several inhibitors under development. Given the negative regulatory role of CBL-B on the immune system, various CBL-B inhibitors have shown the potential to enhance anti-tumor immunity, indicating their potential as a method to overcome immunotherapy resistance.

NX-1607 is a CBL-B inhibitor that inhibits tumor growth in mouse tumor models and promotes the infiltration of NK cells and activated CD8^+^ T cells into the tumor microenvironment. When combined with immune checkpoint inhibitors, NX-1607 further enhances anti-tumor activity and prolongs the survival of tumor-bearing mice [202]. NX-1607 is currently undergoing a Phase I clinical trial (NCT05107674) to evaluate its preliminary efficacy in advanced and treatment-resistant malignant tumors [203].

In an ongoing clinical trial (NCT05662397), the CBL-B inhibitor HST-1011 is being evaluated for its efficacy, both as a monotherapy and in combination with anti-PD-1 antibodies, in patients with tumors that are refractory or resistant to immune checkpoint inhibitors [204]. Another CBL-B inhibitor, ZM-8026, has shown anti-tumor efficacy both as a monotherapy and in combination with PD-1 antibodies. Additionally, ZM-8026 maintains CD8^+^ T cell proliferation and function in an immunosuppressive microenvironment. Treatment with ZM-8026 can induce anti-tumor immune memory and increase the activation of NK cells and macrophages in mouse models [205]. In preclinical tumor models, the CBL-B inhibitor YF550-C1 enhanced the function of CD8^+^ T cells and NK cells. The combination of YF550-C1 and PD-1 antibodies exhibited anti-tumor effects. Interestingly, YF550-C1 alone was able to inhibit tumor progression in PD-1 antibody-resistant NSCLC tumor models, a phenomenon that warrants further investigation through clinical trials [206].

In addition to E3 ubiquitin ligases, deubiquitinating enzymes (DUBs), such as ubiquitin-specific proteases (USPs), are increasingly becoming targets for cancer therapy. Numerous inhibitors targeting these USPs have already been developed.

Almac4 is a USP7 inhibitor that induces the expression of P53 in tumor cells in a dose-dependent manner, thereby inhibiting tumor cell proliferation. Additionally, it suppresses the expression of PD-L1 in tumor cells, enhancing anti-tumor immunity [119]. Other USP7 inhibitors, such as P22077, X36, YCH2823, and FT-671, have all demonstrated anti-tumor activity and are currently in preclinical trial stages [207–210].

Not only USP7 inhibitors but also USP8 inhibitors are involved in regulating anti-tumor immunity. Studies have shown that the USP8 inhibitor DUBs-IN-2 enhances MHC-I antigen presentation, promoting CD8^+^ T cell-mediated tumor killing. The combination of DUBs-IN-2 and anti-PD-1 antibodies significantly improves the survival rate of tumor-bearing mice [112]. Another USP8 inhibitor, DC-U4106, has also shown anti-tumor activity in xenograft tumor models and is currently in the preclinical stage [211].

All the mentioned compounds and their research progress have been summarized in Table 3. In summary, although drugs targeting ubiquitin signaling are not yet widely used in clinical cancer therapy, preliminary data from mouse models and clinical trials suggest that this strategy holds potential for treating cancer and overcoming resistance to immunotherapy. Future research should focus on improving the pharmacokinetic properties of drugs, innovating delivery methods and formulations, designing more selective inhibitors, and exploring the potential of combining small molecule inhibitors with gene therapy or adoptive cell therapy.

**Table 3:** Representative drugs targeting ubiquitin signaling and their research progress

Drugs	Targets	Status	Reference
BI-907,828	MDM2	Phase II	[189]
BI-907,828	MDM2	Phase I	[190]
BI-907,828	MDM2	Phase III	[191]
APG-115	MDM2	Phase II	[194]
APG-115	MDM2	Phase I	[195]
AMG-232	MDM2	Preclinical	[196]
ASTX295	MDM2	Phase I	[198]
NVP-HDM201	MDM2	Phase I	[199]
NX-1607	CBL-B	Phase I	[203]
HST-1011	CBL-B	Phase I/II	[204]
ZM-8026	CBL-B	Preclinical	[205]
YF550-C1	CBL-B	Preclinical	[206]
P22077	USP7	Preclinical	[207]
X36	USP7	Preclinical	[208]
YCH2823	USP7	Preclinical	[209]
FT-671	USP7	Preclinical	[210]
DC-U4106	USP8	Preclinical	[211]

## Conclusions and perspectives

Recently, drugs targeting ubiquitination modification have shown preliminary anti-tumor effects in clinical trials. For instance, combining the cereblon E3 ubiquitin ligase modulator Mezigdomide with dexamethasone has shown therapeutic promise in the treatment of multiple myeloma (NCT03374085) [212]. Additionally, via the ubiquitin-proteasome pathway, the antibody-drug conjugate polatuzumab vedotin enables MCL-1 degradation. Its combination with the BCL-2 inhibitor venetoclax and anti-CD20 antibodies exhibits potential in treating relapsed or refractory lymphoid neoplasms (NCT02611323) [213]. Many other cases demonstrating the enhancement of immune checkpoint inhibitor efficacy through targeting ubiquitination modification pathways are also highlighted in this review.

The activity of immune cells and tumor cells; extracellular chemokines; tumor antigen presentation; immune checkpoints; and the tumor immune microenvironment are all critical factors influencing the efficacy of immunotherapy, with ubiquitination modification playing a wide-ranging regulatory role in these processes. Targeting ubiquitination modification as a therapeutic strategy offers several advantages: it extensively regulates cell signaling transduction with a large number of substrates; it precisely controls substrate protein degradation through protein-protein interactions; it modulates protein function through different ubiquitin chain linkage mechanisms; and it provides a potential means of regulating the degradation of difficult-to-target proteins. This review proposes several potential strategies for increasing the effectiveness of immunotherapy by targeting ubiquitination modification. These include: inhibiting immunotherapy-resistant signaling pathways like WNT/β-catenin; promoting the anti-tumor impact of interferons; inhibiting the tumor immune evasion tactics mediated by cytokines; improving tumor antigen presentation by modulating MHC signaling pathways; strengthening the activity of anti-tumor immune checkpoints while inhibiting those associated with tumor immune evasion; counteracting the immunosuppressive tumor microenvironment shaped by MDSCs, TAMs, and Tregs; increasing tumor immunogenicity through mechanisms such as ferroptosis and pyroptosis; and enhancing the effectiveness of CAR-T cells.

Future research should not only focus on whether there are new ubiquitination modification targets in these pathways that promote immunotherapy but also further investigate and expand the mechanisms that enhance immunotherapy efficacy. Recent research has demonstrated that, under the regulation of ubiquitination modification, the cGAS-STING signaling pathway enhances the effectiveness of immunotherapy [214]. Enhancing anti-tumor immunity through the targeting of cGAS-STING could offer a theoretical foundation for novel treatment approaches. Additionally, tumors of varied types manifest distinct profiles of gene expression and the activation of signaling pathways, which may suggest that precise tumor typing based on tumor cell characteristics and regulation of key oncogenic signaling pathways through ubiquitination modification could also be a potential approach to improving immunotherapy efficacy. In conclusion, ubiquitination modification plays a crucial regulatory role in immunotherapy, offering a new approach to overcoming resistance.

## Data Availability

No datasets were generated or analysed during the current study.
